# Distinct developmental and degenerative functions of SARM1 require NAD^+^ hydrolase activity

**DOI:** 10.1371/journal.pgen.1010246

**Published:** 2022-06-23

**Authors:** E. J. Brace, Kow Essuman, Xianrong Mao, John Palucki, Yo Sasaki, Jeff Milbrandt, Aaron DiAntonio

**Affiliations:** 1 Department of Developmental Biology, Washington University School of Medicine, St. Louis, Missouri, United States of America; 2 Department of Genetics, Washington University School of Medicine, St. Louis, Missouri, United States of America; 3 Needleman Center for Neurometabolism and Axonal Therapeutics, Washington University School of Medicine, St. Louis, Missouri, United States of America; National University of Singapore, SINGAPORE

## Abstract

SARM1 is the founding member of the TIR-domain family of NAD^+^ hydrolases and the central executioner of pathological axon degeneration. SARM1-dependent degeneration requires NAD^+^ hydrolysis. Prior to the discovery that SARM1 is an enzyme, SARM1 was studied as a TIR-domain adaptor protein with non-degenerative signaling roles in innate immunity and invertebrate neurodevelopment, including at the *Drosophila* neuromuscular junction (NMJ). Here we explore whether the NADase activity of SARM1 also contributes to developmental signaling. We developed transgenic *Drosophila lines* that express SARM1 variants with normal, deficient, and enhanced NADase activity and tested their function in NMJ development. We find that NMJ overgrowth scales with the amount of NADase activity, suggesting an instructive role for NAD^+^ hydrolysis in this developmental signaling pathway. While degenerative and developmental SARM1 signaling share a requirement for NAD^+^ hydrolysis, we demonstrate that these signals use distinct upstream and downstream mechanisms. These results identify SARM1-dependent NAD^+^ hydrolysis as a heretofore unappreciated component of developmental signaling. SARM1 now joins sirtuins and Parps as enzymes that regulate signal transduction pathways via mechanisms that involve NAD^+^ cleavage, greatly expanding the potential scope of SARM1 TIR NADase functions.

## Author summary

SARM1 is the central executioner of axon loss, and inhibition of SARM1 is a therapeutic target for many devastating neurodegenerative disorders. SARM1 is the founding member of the TIR-domain family of NAD^+^ cleaving enzymes, destroying the essential metabolite NAD^+^ and inducing an energetic crisis in the axon. This was a surprising finding, as previously studied TIR-domain proteins were characterized as scaffolds that bind signaling proteins to coordinate signal transduction cascades. Indeed, before the discovery of the role of SARM1 in axon degeneration, SARM1 was studied as a regulator of intracellular signaling in immunity and neurodevelopment where it was assumed to act as a scaffold. Here we investigate whether the recently described SARM1 enzymatic activity also regulates such signal transduction pathways. Indeed, we show that a developmental signaling pathway scales with the amount of NADase activity, suggesting an instructive role for NAD^+^ cleavage. While degenerative and developmental SARM1 signaling share a requirement for NAD^+^ cleavage, they utilize distinct upstream and downstream mechanisms. With these findings, SARM1 now joins sirtuins and Parps as enzymes that regulate signal transduction pathways via mechanisms that involve NAD^+^ cleavage, greatly expanding the potential scope of SARM1 TIR NADase functions.

## Introduction

SARM1 (Sterile Alpha and TIR Motif Containing 1) is the central executioner of pathological axon degeneration [1,2]. Loss of SARM1 blocks axon loss after axotomy [3,4]. Activation of SARM1 leads to catastrophic NAD^+^ depletion, inducing a stereoptyped sequences of events resulting in metabolic failure and axon fragmentation [5–7]. The absence of SARM1 is protective in human neurons [8], as well as numerous *in vivo* mouse models of neurodegeneration [9] including chemotherapy-induced peripheral neuropathy [10,11], diabetic neuropathy [12,13], traumatic brain injury [14–16], glaucoma [17], and at least two forms of retinal degeneration [18,19]. Moreover, activating mutations in SARM1 are enriched in patients with ALS [20,21]. SARM1 is a multidomain protein comprised of an autoinhibitory N-terminal ARM/Armadillo repeat containing domain, twin Sterile alpha motif (SAM) domains that mediate multimerization, and an executioner Toll-Interleukin Receptor homology domain (TIR). While TIR domains are best studied as scaffolds [22,23], we discovered that the TIR domain of SARM1 is an NAD^+^ hydrolase (NADase) [7]. Moreover, SARM1 is the founding member of a family of TIR NADases active in animals, plants, bacteria, and archaea [7,24–26]. Hence, NAD^+^ hydrolase activity is likely the primordial function of TIR domains.

While recent interest in SARM1 has focused on its role in neurodegeneration and function as an NADase, SARM1 was originally characterized as a TIR adaptor protein with functions in mammalian innate immunity [27] and invertebrate neurodevelopment and immunity [28–30]. For example, the SARM1 ortholog in *C*. *elegans*, tir-1, specifies asymmetric odorant receptor expression by regulating an ASK1 MAPKKK signaling pathway [29]. In *Drosophila*, neuronal dSarm1 regulates NMJ development [30], while glial dSarm1 promotes phagocytosis of neurons undergoing developmental apoptosis [31]. While these studies established the role of SARM1 orthologs in developmental signaling, work in *Drosophila* was the first to demonstrate that SARM1 is also required for pathological axon degeneration [3]. Moreover, dSarm1 is an NADase [7] and this enzymatic activity is necessary for axon degeneration in the fly [32]. With these dual functions, *Drosophila* is an excellent system to explore the potential interplay between SARM1 as a signaling molecule and SARM1 as an NAD^+^ hydrolase. Might SARM1 NADase activity not only promote degeneration, but also regulate non-degenerative signal transduction?

To test the idea that SARM1 NADase activity regulates signal transduction, we investigated the impact of dSarm1 NADase activity on the development of the fly NMJ. We identified mutants that either enhanced or eliminated dSarm1 NADase activity. These mutations enabled the creation of transgenic fly lines expressing either enzymatically dead (dSarm1^ED^) or catalytically enhanced (dSarm1^CE^) dSarm1 NADase activity. In loss of function studies we found that the NADase activity is essential for dSarm1 to rescue developmental lethality of *dSarm1* mutants. Moreover, in the absence of NADase activity dSarm1 does not promote NMJ growth, and conversely, enhanced NADase activity leads to dramatic NMJ overgrowth. This dSarm1 developmental activity is mediated through a canonical intracellular signaling pathway that includes the MAPKKK Ask1 and the transcription factor Fos. Hence, dSarm1 NADase activity not only promotes axon degeneration [32], but also regulates developmental signal transduction. While NADase activity is required for both the degenerative and signaling roles of dSarm1, we find evidence for different upstream activating and downstream effector mechanisms for these two distinct roles of dSarm1. For example, overexpression of NMNAT1 keeps dSarm1 off during injury signaling, however it does not inhibit NMJ overgrowth due to catalytically enhanced dSarm1^CE^. Downstream of dSarm1, Ask1 is essential for NMJ overgrowth, but its loss has no observable impact on axon degeneration. Similarly, injury can activate human SARM1 to induce axon degeneration in *Drosophila* dSarm1 knockdown mutants, but during *Drosophila* development human SARM1 is incapable of promoting NMJ overgrowth, likely due to a failure to activate human SARM1. Hence, degeneration and development share a requirement for SARM1 NAD^+^ hydrolysis, but utilize distinct upstream and downstream mechanisms. Taken together, these findings demonstrate a novel role for dSarm1 NADase activity in regulating intracellular signaling and suggest that dSarm1-dependent regulation of NAD^+^ or NAD^+^ break down byproducts regulate signal transduction. The independent companion study from Herrmann et al. uses different genetic tools to assess the role of dSarm1 in developmental glial phagocytosis and comes to very similar conclusions [33].

## Results

### Identification of key residues in the dSarm1 TIR domain that affect NADase activity

**Fig 1:**
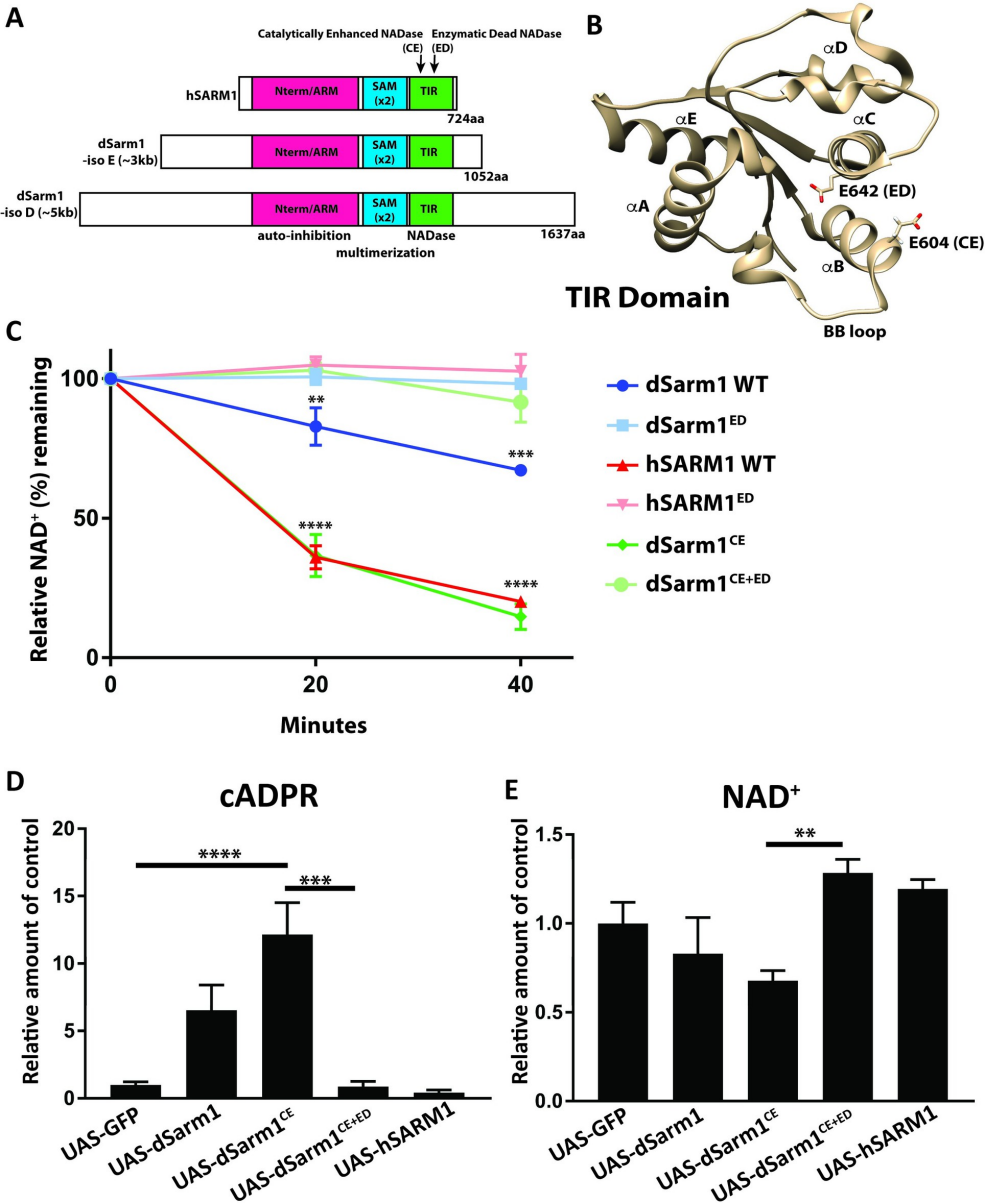
Identification of gain and loss of function mutations in the TIR domain of dSarm1. **A,** Schematic of the major domains and location of the point mutations that confer either catalytically enhanced or enzymatic dead NADase activity on dSarm1. Human SARM1 and isoforms E (3kb) and D (5kb) of *Drosophila* Sarm1 are depicted **B,** Crystal structure of the TIR domain from human SARM1 is depicted (PDB structure 6O0Q; from [24]) with mutant residues depicted. **C,** NADase activity assay. One hundred nanograms of TIR proteins purified from HEK cells were incubated with NAD^+^ for indicated times. NAD^+^ metabolites were quantified by HPLC and percent of NAD^+^ remaining is shown. Values are presented as mean +/- SEM. Statistics were obtained by performing a one-way ANOVA with Tukey’s multiple comparisons for genotypes. (n = 3). (**p<0.01, ***p<0.001, ****p<0.0001) **D,** Transgenes (isoform E– 3kb) were expressed under control of the pan-neuronal driver BG380-Gal4. The ventral nerve cord from third instar larva expressing individual transgenes were dissected and metabolites were extracted for mass spectrometry. cADPR levels were measured as a biomarker for Sarm1 NADase activity. The number of independent pooled samples are (n = 9, 5, 8, 5, and 4 respectively) for each genotype. Each sample (n = 1) is from an independent pool of 5 ventral nerve cords (***p<0.001, ****p<0.0001). Values are presented as mean +/- SEM. Statistics were obtained by performing a one-way ANOVA with Tukey’s multiple comparisons for genotypes. **E,** NAD^+^ levels were measured from the identical samples as Fig 1D (**p<0.01). Statistics were performed as in 1D.

To test the role of dSarm1 NADase activity for cell signaling, we set out to develop complementary mutations in the TIR domain that resulted in either inactive or enhanced NADase activity. In human SARM1 the glutamate at position 642 is required for NAD^+^ hydrolysis and an E_642_A mutation in human SARM1 abrogates NADase activity [7]. We mutated the orthologous glutamate in the *Drosophila* TIR domain (E_893_A in isoform E-3kb), purified wild-type and E_893_A mutant *Drosophila* TIR protein (dTIR) from HEK293 cells, and assessed consumption of NAD^+^ in an *in vitro* enzymatic assay. In this biochemical assay, SARM1 is active without the need for injury to trigger activation. As predicted, mutation of this glutamate abrogates the NADase activity of the *Drosophila* TIR domain and is referred to throughout as the “Enzymatic Dead (ED)” NADase (Fig 1).

In agreement with previous findings [7], dTIR was significantly slower in processing NAD^+^ than human TIR (hTIR) (Fig 1C). We hypothesized that to increase the NADase activity of dSarm1 we could swap residues in dSarm1 to the corresponding residues from hSARM1. We identified a single point mutation (fly D_855_E in isoform E-3kb, equivalent to human E_604_) that confers enhanced NADase activity to dTIR and that is similar in activity to hTIR (Fig 1). We term dSarm1 variants with a D to E mutation at this site to be “catalytically enhanced (CE).” This enhanced NADase activity is fully dependent on the catalytic activity of dSarm1 since a double mutant incorporating both the CE variant (D_855_E) and the ED variant has no NADase activity (Fig 1C). Identification of these catalytically enhancing and enzymatic dead variants allowed us to test the role of NADase activity for the function of full-length dSarm1.

We created a set of full-length dSarm1 transgenic animals to test the consequence of altering the NADase activity of dSarm1 *in vivo*. The *dSarm1* genomic locus encodes eight protein isoforms. Of the isoforms, some are larger proteins with N- and C-terminal extensions that are not homologous to mammalian SARM1, while others are shorter and are more like mammalian SARM1. One of the larger isoforms (isoform D) is sufficient to rescue lethality of *dSarm1* mutants [3], but the role of smaller isoforms has not been tested. We generated transgenes expressing one transcript from both the large and small class (Fig 1A). The full-length coding sequence of *dSarm1* isoform E (3kb) and isoform D (5kb) were tagged with HA at the C-terminus, cloned into pUAST-attB and inserted into the defined genomic landing site ZH-86Fb (BDSC#24749). We also generated the site directed mutants of both isoforms in order to manipulate NADase activity. In total, eight unique *dSarm1* transgenic lines were generated with the two isoforms (for clarity, amino acid number refers to the corresponding amino acid in the human sequence regardless of isoform) a) full-length *dSarm1*, b) *dSarm1*^*ED*^, loss of NADase activity (E_642_A), c) *dSarm1*^*CE*^, catalytically enhanced NADase activity (D_604_E), d) *dSarm1*^*CE+ED*^, double mutant that combines both the catalytically enhanced D_604_E mutation with the E_642_A loss of function mutation. We generated this double mutant to ensure that any gain-of-function phenotype produced by expression of the catalytically enhanced mutation can be attributed to the NADase function of dSarm1 rather than an unappreciated neomorphic effect. In addition, a full-length human *SARM1* (hSARM1) transgene was generated. The nine transgenes were inserted in the same location to minimize variation due to genomic location.

### *dSarm1* encodes a functional NADase *in vivo*

The activity of both dTIR and hTIR can be followed *in vitro* by measuring the levels of cyclic ADPR (cADPR), a cleavage product of the NADase reaction (Essuman et al [7]). Further, cADPR is also a direct biomarker of SARM1 activity *in vivo* in mice [34]. To test whether these engineered dSarm1 variants display NADase activity *in vivo*, we expressed the dSarm1, dSarm1^CE^, and dSarm1^CE+ED^ transgenes (isoform E - 3kb) in neurons under the control of the pan-neuronal BG380-Gal4 driver, collected ventral nerve cords from third instar larvae, and extracted metabolites for mass spectrometry (LC-MS-MS) analysis. Overexpression of wild-type dSarm1 leads to an increase in cADPR levels in the ventral nerve cord (Fig 1D). Expression of dSarm1^CE^ leads to an even greater increase in cADPR levels, and this effect is completely abrogated in the enzymatically dead dSarm1^CE+ED^ double mutant. Moreover, expression of the dSarm1^CE^ transgene leads to a significant drop in NAD^+^ levels compared to the inactive dSarm1^CE+ED^ transgene (Fig 1E). We detect no significant decrease in NAD^+^ levels upon overexpression of wild-type dSarm1, suggesting that NAD^+^ biosynthetic pathways are able to compensate for the consumption of NAD^+^ in this case. Moreover, we are measuring NAD^+^ from the entire nervous system, but only expressing dSarm1 transgenes in neurons, and so any change in measured NAD^+^ levels will be blunted by the presence of glia and other non-neuronal cells. We also detect no loss of NAD^+^ or increase in cADPR upon expression of human SARM1. This suggests that when hSARM1 is expressed in *Drosophila* neurons it is not active, in contrast to dSARM1 which is active. Taken together, these studies are the first direct demonstration that dSarm1 is a functional NADase *in vivo*. Hence, these engineered transgenes provide the opportunity to assess the function of dSarm1 protein with either enhanced or disrupted NADase activity.

### Expression of dSarm1 transgenes

Before comparing the function of our engineered dSarm1 variants, we first assessed their expression. All eight dSarm1 transgenes and the hSARM1 transgene were expressed in the nervous system under the control of *dVGlut*-Gal4 driver and protein localization and levels were assessed in peripheral nerves of third instar larvae by immunofluorescence (HA-tag). Protein levels of each isoform was quite different, with the larger isoform D (5kb) present at lower levels than isoform E (3kb). However, as we will demonstrate below, the two isoforms performed similarly in our initial functional assays and therefore we concentrated on the smaller isoform E (3kb) for most experiments. Within each isoform, there were no significant difference in expression of the catalytically enhanced (CE) and wild-type transgenes (S1 Fig). Hence, any difference in phenotype induced by the site-directed mutants can be attributed to differences in their NADase activity rather than secondary effects of differing protein levels.

### The NADase function of dSarm1 is required for viability

With a range of validated full-length dSarm1 transgenes in hand, we tested which dSarm1 variants could perform the essential functions of endogenous dSarm1 and rescue viability of *dsarm1* mutants [3]. Transgenes were expressed ubiquitously with *daughterless*-Gal4, and tested for their ability to rescue viability of *dsarm1* mutant offspring. As shown in Table 1, expression of wild-type (WT) isoform E (3kb) or D (5kb) resulted in rescue of *dsarm1* mutants, demonstrating that both transgenes are functional. Interestingly, pan-neuronal expression of these dSarm1 transgenes (BG380-Gal4) does not rescue viability, suggesting an essential role for non-neuronal expression of dSarm1.

**Table 1:** Rescue Expression of dSarm1 transgenes rescue lethality of dSarm1 mutants. Daughterless-GAL4 (da-GAL4) was used to drive ubiquitous expression of Sarm1 transgenes. Homozygous dSarm1^4705^/dSarm1^4621^ mutant animals are lethal. Expression of transgenes in the mutant background tests for the ability of the transgene to rescue dSarm1 function. Expression of transgenes in a WT background tests for lethality of the transgene itself as is seen in the case of dSarm1^CE^. Quantification is shown for each genotype as two sets of numbers 1) number of rescued mutant flies relative to control flies and 2) WT flies expressing transgene versus control flies). Quantification is as follows: da-GAL4/+ (0 mutant+transgene: 220 control and 144 WT+transgene: 66 control); da-GAL4/ dSarm1 (3KB) (166:73 and 171:159); da-GAL4/dSarm1^ED^ (3KB) (0:239 and 191:182); dSarm1^CE^ (3KB) (0:251 and 0:262); da-GAL4/dSarm1^CE+ED^ (3KB) (0:201 and 225:185); da-GAL4/dSarm1 (5KB) (204:63 and 90:243); da-GAL4/dSarm1^ED^ (5KB) (0:187 and 199:90); da-GAL4/dSarm1^CE^ (5KB) (0:164 and 0:195); da-GAL4/dSarm1^CE+ED^ (5KB) (0,153 and 185:206); da-GAL4/human SARM1 (0,370 and 288:196).

	Sarm^4705/4621^	WT
**da-GAL4/+**	lethal	viable
**da-GAL4/UAS-dSarm1 (3KB)**	viable	viable
**da-GAL4/UAS-dSarm1**^**ED**^ **(3KB)**	lethal	viable
**da-GAL4/UAS-dSarm1**^**CE**^ **(3KB)**	lethal	lethal
**da-GAL4/UAS-dSarm1**^**CE+ED**^ **(3KB)**	lethal	viable
**da-GAL4/UAS-dSarm1 (5KB)**	viable	viable
**da-GAL4/UAS-dSarm1**^**ED**^ **(5KB)**	lethal	viable
**da-GAL4/UAS-dSarm1**^**CE**^ **(5KB)**	lethal	lethal
**da-GAL4/UAS-dSarm1**^**CE+ED**^ **(5KB)**	lethal	viable
**da-GAL4/UAS-human SARM1**	lethal	viable

We next tested the site-directed mutants of dSarm1. First, we find neither isoform D (5kb) nor E (3kb) dSarm1^ED^ transgenes could rescue lethality. Hence, the NADase function of dSarm1 is necessary for the essential roles of dSarm1 during development, and so may play roles beyond its well-characterized function in axon degeneration[7,32]. Next, we tested whether the catalytically enhanced dSarm1^CE^ (D_604_E) mutant could rescue the lethality of *dsarm1* mutants. Expression of dSarm1^CE^ does not rescue the lethality of a d*sarm1* mutant, however, the lack of rescue is likely not due to loss-of-function, but instead to gain-of-function lethality. Indeed, expression of either isoform of dSarm1^CE^ induces lethality in an otherwise wild-type background, consistent with the known degenerative effects of active SARM1 [4,6,35]. The lethality is dependent on NADase activity rather than an unrelated neomorphic effect since the double mutant dSarm1^CE+ED^ transgene does not lead to lethality. Selective expression of dSarm1^CE^ in many different cell types including neurons or glia also induces lethality (selective Gal4 lines listed in Materials and Methods). Taken together, these findings demonstrate that a) ubiquitous expression of full-length dSarm1 rescues *dsarm1* lethality, b) that the substantial differences between isoforms D and E are not essential for their function in survival, c) that the NADase activity of dSarm1 is essential for survival, and that d) dSarm1 with enhanced NADase activity induces lethality when expressed ubiquitously. This set of transgenes now allows us to test the role of SARM1-dependent NADase activity on cellular signal transduction pathways.

### The NADase activity of dSarm1 modulates NMJ growth

**Fig 2:**
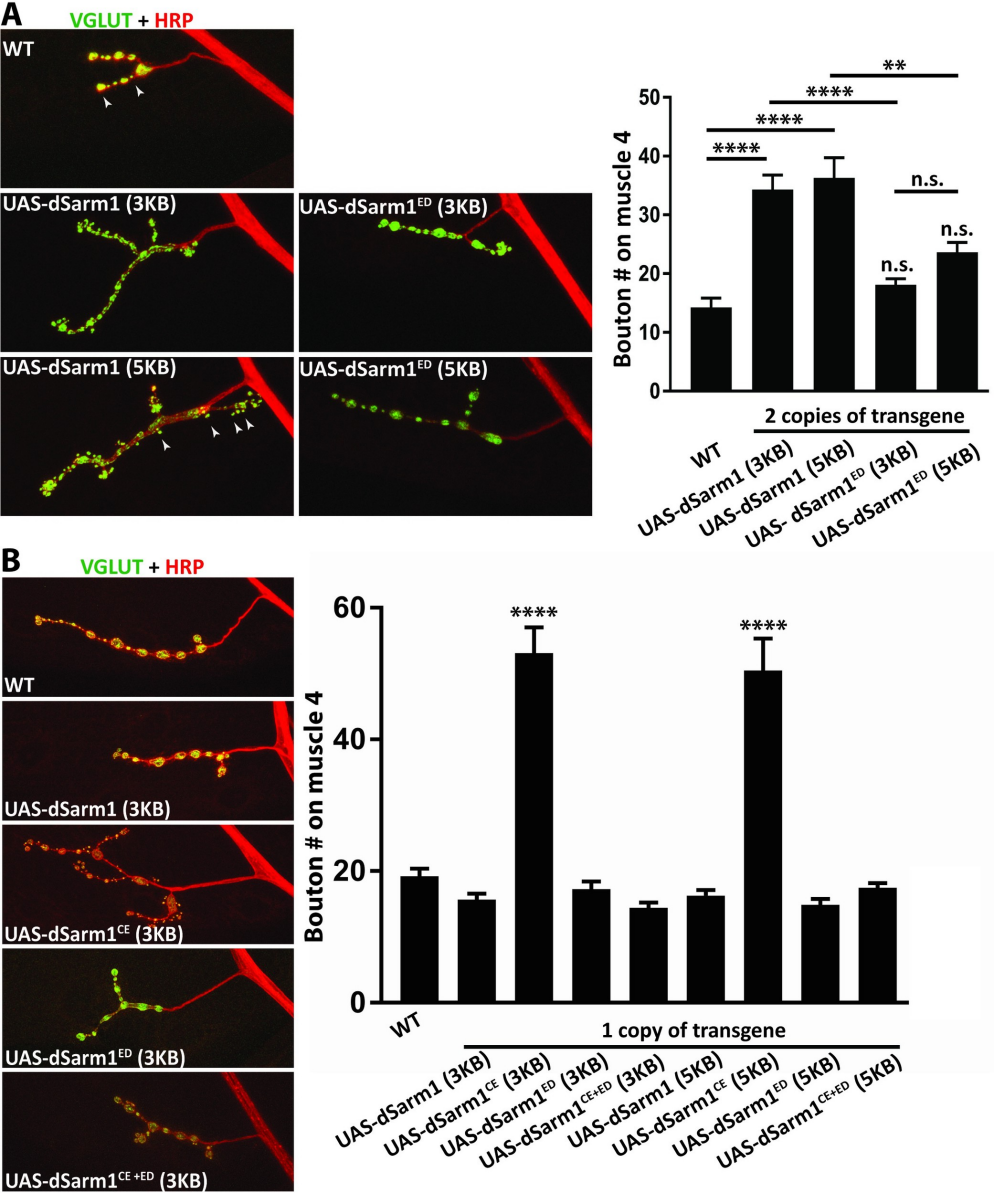
The NADase activity of dSarm1 is important for regulating NMJ growth. ***A***, Overexpression (*dVGlut*-Gal4) of dSarm1 leads to overgrowth of NMJ and requires the NADase activity. Representative confocal images of muscle 4 synapses from third instar larva co-stained for presynaptic dVGlut (green) and neuronal HRP (Red). Representative boutons depicted with arrowheads. Genotypes of the progeny are listed in the figure. Genotypes in Fig 2 A are homozygous, therefore two copies of the dSarm1 transgene are present. Quantification of bouton number is from motoneuron MN4b on muscle 4 of male progeny (14.3, 34.3, 36.3, 18.1, 23.7 boutons, respectively) (n = 12, 20, 16, 20, 20). Values are presented as mean +/- SEM. Statistics were obtained by performing a one-way ANOVA with Tukey’s multiple comparisons for genotypes. There is a significant increase (****p<0.0001) in the number of boutons between WT and progeny expressing two copies of dSarm1 (both isoform E- 3kb and isoform D-5kb). Overexpression of mutant forms of dSarm1^ED^ (lacking NADase activity) do not lead to NMJ overgrowth. Example boutons are depicted by white arrowheads. (**p<0.01). ***B*,** The catalytically enhanced NADase activity of dSarm1^CE^ leads to robust NMJ overgrowth. Images and immunostaining are similar to above. Genotypes of the progeny are listed in the figure, however, only a single copy of each transgene is present. Quantification of the bouton number from motoneuron MN4b on muscle 4 of male progeny (19.2, 15.7, 53.1, 17.3, 14.4, 16.3, 50.5, 14.9, 17.5 boutons, respectively) (n = 30, 28, 27, 27, 26,27, 28, 25, 31). Values are presented as mean +/- SEM. Statistics were performed as in 2A. There is a significant increase (****p<0.0001) in the number of boutons between dSarm1 overexpression and dSarm1^CE^ overexpression.

dSarm1 regulates development of the fly NMJ and overexpression of dSarm1 leads to modest synaptic terminal overgrowth [30]. We used this system to test the role of NADase activity in dSarm1-dependent modulation of NMJ growth. We first attempted to replicate the prior findings of NMJ overgrowth upon overexpression of dSarm1. We observe an increase in the number of synaptic boutons at the NMJ on muscle 4 when either isoform of wild-type dSarm1 is expressed from the motor neuron driver *dVGlut*-Gal4 (Fig 2A). This modest overgrowth required two copies of the transgene, likely due to low expression of the transgene from this genomic locus (Fig 2B). We next asked whether this overgrowth required the NADase activity of dSarm1 by expressing two copies of the enzymatic mutant E_642_A (dSarm1^ED^). Interestingly, expression of dSarm1^ED^ did not lead to overgrowth, indicating that the NADase activity of dSarm1 is necessary for NMJ overgrowth (Fig 2A). To test whether NADase activity is not only permissive for NMJ growth but also instructive, we investigated whether enhancing NADase activity would further increase NMJ overgrowth. We expressed dSarm1^CE^ from *dVGlut*-Gal4 and counted the number of synaptic boutons on muscle 4 NMJ. This was first attempted with two copies of the transgene as with wild-type dSarm1, but strong expression of dSarm1^CE^ early in development is deleterious leading to synaptic retraction. Therefore, single copies of each of the transgenes were expressed and the size of the NMJ was measured. Expression of dSarm1^CE^ resulted in robust NMJ overgrowth compared to wild-type dSarm1, and this phenotype requires NADase activity of dSarm1 since there is no overgrowth when the enzymatic dead double mutant dSarm1^CE+ED^ is expressed (Fig 2B). While quantification was performed at the muscle 4 synapse, the overgrowth phenotype was consistent across NMJs at many muscles. Hence, NMJ growth scales with the level of enzyme activity, demonstrating that the NADase activity of dSarm1 is capable of regulating developmental signaling and so has a function beyond catastrophic NAD^+^ collapse and axon degeneration.

### dSarm1 signals through an Ask1- and Fos-dependent transcriptional program to regulate NMJ growth

**Fig 3:**
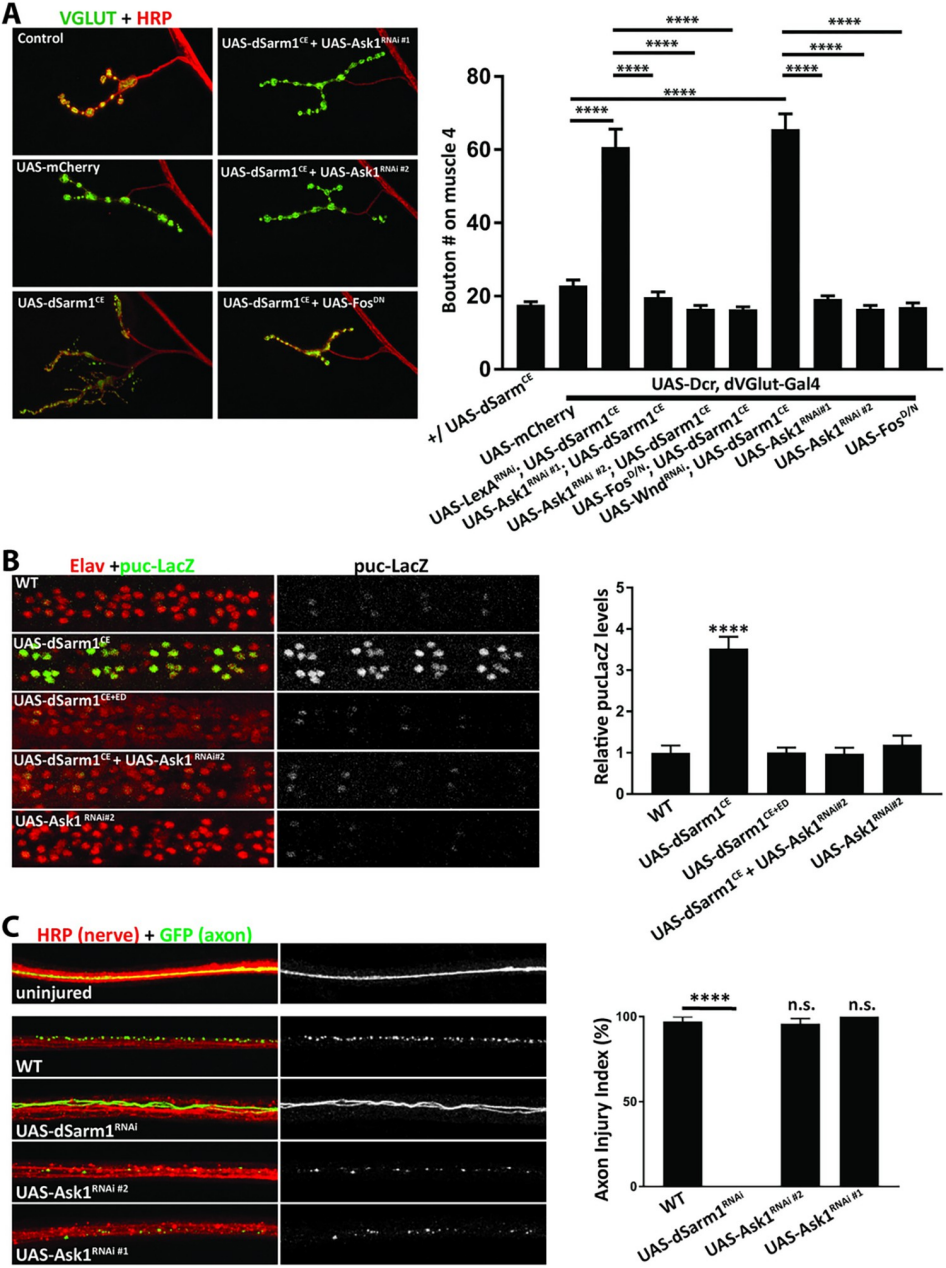
The Ask1 MAPK pathway functions downstream of dSarm1 to regulate NMJ growth, but not axonal injury. ***A*,** Overexpression of dSarm1^CE^ (isoform E-3kb) results in robust overgrowth at the NMJ. Overgrowth is suppressed by expression of either of two non-overlapping Ask1 RNAi transgenes or Fos^D/N^. Expression of Ask1 RNAi or Fos^D/N^ do not lead to phenotypes on their own. Representative confocal images of muscle 4 synapses from third instar larva costained for presynaptic DVGLUT (green) and neuronal HRP (Red). Genotypes of the progeny are listed: UAS-Ask1^RNAi #1^ = KK110228, UAS-Ask1^RNAi #2^ = 35331, UAS-Wnd^RNAi^ = 103410, UAS-LexA^RNAi^ = 67947, UAS-dSarm1^RNAi^ = KK105369. Quantification of the bouton number from motoneuron MN4b on muscle 4 of male progeny (17.6, 22.9, 60.8, 19.8, 16.6, 16.4, 65.6, 19.2, 16.5, 17.1, respectively). For Fig 3A-C, values are presented as mean +/- SEM. Statistics were obtained by performing a one-way ANOVA with Tukey’s multiple comparisons for genotypes. (****p<0.0001). (n = 23, 27, 22, 30, 25, 34, 28, 36, 30, 31). ***B*,** The Jnk phosphatase *puckered (puc)* is a transcriptional readout of the Jnk signaling pathway. Neuronal expression (*BG380-gal4*) of dSarm1^CE^ (isoform E-3kb) in neurons results in a 3.5-fold increase in *puc-lacZ* expression in neurons along the dorsal midline of the ventral nerve cord compared with WT controls. There is no increase in *puc-LacZ* levels in the double mutant, dSarm1^CE+ED^ demonstrating a requirement for the NADase activiy of dSarm1. Furthermore, knockdown of Ask1 leads to full suppression of the increase in *puc-lacZ* expression. Quantification of *puc-lacZ* levels normalized from male progeny (1.00, 3.53, 1.01, 0.97, 1.20, respectively). (****p<0.0001). (n = 7, 5,5,7,7) ***C*.,** The segmental nerves of third instar larva are stained by HRP (red) and single axons are labeled by the expression of UAS-mCD8:GFP by the M12-Gal4 driver. In wild-type larva, axons distal to the crush site are extensively fragmented by 18 h after injury as seen by fragmentation of the GFP-labeled axon. Knockdown of dSarm1 results in pronounced axonal protection (****p<0.0001) in distal axons after injury. Conversely, knockdown of Ask1 shows no axonal protection. Axons from female progeny were scored by researcher blind to genotype using a scale ranging from completely continuous (0%), to continuous with varicosities (25%), to partially discontinuous (50%), to mostly fragmented with a few segments of continuity (75%), to completely fragmented (100%) [38]. (n = 9, 18, 18,11).

Having demonstrated that the dSarm1 NADase regulates signaling, we next addressed the identity of the downstream signaling pathway. In addition to the synaptic overgrowth observed in dSarm1^CE^ animals, we also observed smaller synaptic boutons and less DVGLUT protein at the NMJ. These are hallmark phenotypes of excess JNK signaling [36–38]. SARM1 orthologs can regulate JNK kinase signaling [29,39], and others and we previously demonstrated that activation of the JNK pathway in *Drosophila* motor neurons promotes synaptic growth [36,40]. This synaptic growth pathway includes the MAP3K DLK/Wallenda that functions through JNK and the transcription factor Fos to promote synaptic growth [36,41]. In worms, however, SARM1 functions through the MAP3K Ask1 [29,32]. Hence, we tested both JNK-activating MAP3Ks (DLK/Wallenda and Ask1) and the JNK-regulated transcription factor Fos. We reasoned that if dSarm1 NADase activity influences one of these signaling proteins, then inhibition of the relevant protein should suppress the dSarm1-dependent synaptic overgrowth phenotype. First, we expressed a previously validated [42] transgenic RNAi construct targeting Wallenda (DLK) in motor neurons while simultaneously overexpressing dSarm1^CE^. Knockdown of Wallenda fails to suppress the NMJ overgrowth induced by expression of this catalytically-enhanced dSarm1^CE^ transgene (Fig 3A). In contrast, either of two non-overlapping RNAi transgenes targeting Ask1 fully suppresses dSarm1-dependent NMJ overgrowth yet have no impact on NMJ growth on their own in a wild-type background (Fig 3A). Hence, Ask1 is the relevant kinase downstream of dSarm1 for synaptic terminal overgrowth.

Activation of MAP kinase stress pathways can induce a transcriptional program that leads to NMJ overgrowth in *Drosophila* [43], and so we investigated whether SARM1 signaling activates such a transcriptional program. Ask1 signals through the MAP Kinase Jnk, and Jnk signaling upregulates expression of the *Puckered* gene. A LacZ enhancer trap under the control of the endogenos *Puckered* promoter is a readout of *Puckered* expression and, hence, JNK signaling [44]. We measured the intensity of LacZ staining in neuronal cell bodies in third instar larva. Expression of UAS-dSarm1^CE^ leads to an ~3.5 fold increase in LacZ staining intensity compared to WT controls (Fig 3B). This increase was dependent on Ask1 as both non-overlapping RNAi transgenes targeting Ask1 fully suppressed the increase in LacZ staining (Fig 3B) demonstrating that dSarm1 activates a transcriptional response downstream of Ask1.

We next investigated the identity of the downstream transcription factor regulating NMJ growth. We previously demonstrated that the Fos transcription factor is required for NMJ overgrowth downstream of the MAPKKK Wallenda [36]. Similarly, expression of a dominant negative transgene targeting Fos fully suppressed the Ask1-dependent NMJ overgrowth induced by expression of dSarm1^CE^ (Fig 3A). Hence, catalytically-enhanced dSarm1 activates a MAPK signaling pathway that shares the downstream Fos transcriptional program with the well-known Highwire synaptic growth program [36,45] while using the MAP3K Ask1 rather than DLK/Wallenda as the MAP3K. These findings therefore unify the original studies identifying a SARM1/ASK1 pathway with the recent discovery that SARM1 is an NADase into a single signaling pathway.

### Distinct downstream mechanisms regulate dSarm1 function in developmental and degenerative signaling

SARM1 NADase activity is required for both axon degeneration [1,7] and to promote NMJ growth. Since Ask1 functions downstream of dSarm1 NADase activity in regulating NMJ growth, we investigated whether Ask1 might also function downstream of SARM1 in either *Drosophila* or mammalian models of axon degeneration. In *Drosophila*, crushing the segmental nerves induces Wallerian degeneration of the distal axon [46]. In wild-type animals, GFP-labeled motor axons are completely fragmented by 18 h post-injury (Fig 3C). This degeneration is fully dSarm1-dependent, as neuronal expression of an RNAi targeting dSarm1 provides complete axonal protection following this crush injury. In contrast, expressing either of the two Ask1 RNAi constructs that fully suppressed dSarm1-dependent NMJ overgrowth provided no axonal protection (Fig 3C). In complementary studies, we tested whether Ask1 participates in axon degeneration in mammals. Cultured mouse embryonic dorsal root ganglion (DRG) neurons grow long axons that undergo mSARM1-dependent axon degeneration following axotomy [3,4]. To test the role of ASK1 in mammalian axons, we treated DRG neurons with the well-validated ASK1 inhibitor Selonsertib (GS-4997, [47,48]) for either 1 h or 24 h prior to injury. Neither dosing regimen slowed axon degeneration after axotomy (S2 Fig). These studies demonstrate that Ask1 does not promote axonal degeneration in either *Drosophila* or mammalian models. Hence, distinct mechanisms function downstream of SARM1 NADase activity in developmental and degenerative contexts.

### Distinct upstream mechanisms regulate dSarm1 activation for developmental and degenerative signaling

We recently defined the molecular mechanism of injury-induced SARM1 activation, demonstrating that SARM1 is a metabolic sensor that responds to a rise in the ratio of the NAD^+^ precursor NMN to the level of NAD^+^ (NMN/NAD^+^ ratio) [49]. This mechanism explains how mammalian SARM1 is activated by the loss of the NAD^+^ biosynthetic enzyme NMNAT2 that occurs upon axon injury [50] defining how NMNAT2 acts as an upstream negative regulator of SARM1. In *Drosophila*, the endogenous dNmnat is also a labile protein that is lost after axon injury [45,51]. Here we investigate whether activation of dSarm1 for developmental signaling works through the same molecular mechanism as that activated after injury. In injury signaling, overexpression of NMNAT1 or dNmnat potently blocks axon degeneration in mouse [52] and *Drosophila* [53,54] by maintaining SARM1 in an inactive state [55] via the enzymatic conversion of NMN to NAD^+^, which keeps the ratio of NMN/NAD^+^ near its normal level. Hence, we investigated whether overexpression of NMNAT enzymes blocks the synaptic overgrowth phenotypes induced by neuronal expression of the catalytically enhanced dSarm1^CE^ transgene.

We first tested whether dSarm1^CE^ maintained dNMNAT-dependent regulation. Single motor axons expressing GFP in third instar larvae show continuous and smooth axonal GFP staining. Knockdown of dNMNAT by RNAi in the GFP expressing axons results in axon degeneration, and this is fully SARM1-dependent as homozygous *dsarm1* mutants are resistant to axonal degeneration induced by loss of dNMNAT (S3A Fig). However, reexpression of UAS-dSarm1^CE^ in *dsarm1* mutants restores axonal degeneration upon dNMNAT1 knockdown, demonstrating that dSarm1^CE^ maintains its regulation by dNMNAT (S3A Fig). Importantly, dSarm1^CE^ does not result in spontaneous axon degeneration in the absence of dNMNAT knockdown (S3B Fig).

**Fig 4:**
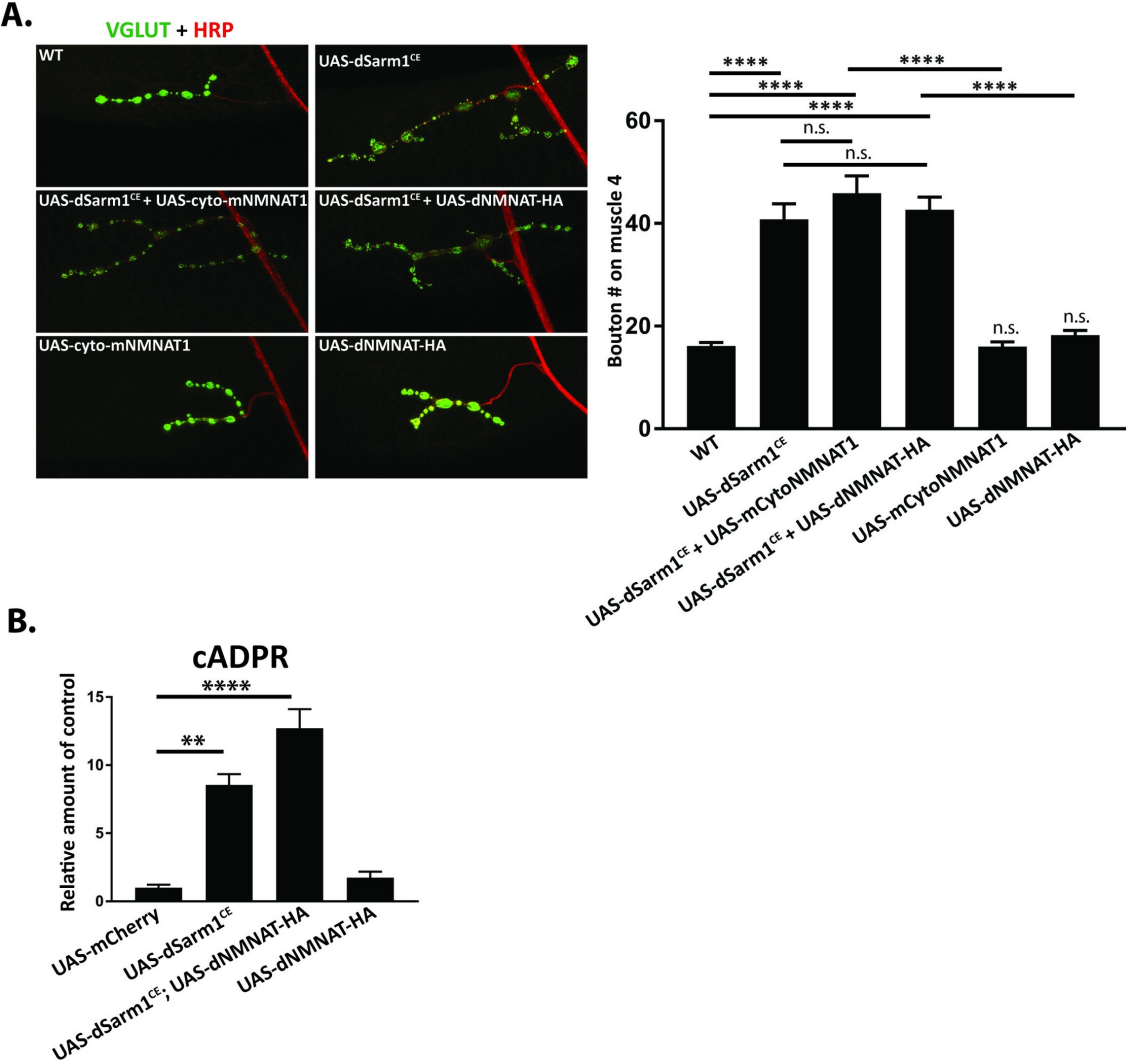
dSarm1 is activated by distinct signals during NMJ growth and in axon injury. Activation of dSarm1 for NMJ growth and axonal injury appears to be unique. ***A*,** In uninjured animals, overexpression (dVGlut-Gal4) of either cyto-mNMNAT1 or dNmnat does not block NMJ overgrowth due to dSarm1^CE^ (isoform E-3kb) overexpression. Representative confocal images of muscle 4 synapses from third instar larva co-stained for presynaptic dVGlut (green) and neuronal HRP (Red). Quantification of the bouton number from motoneuron MN4b on muscle 4 of male progeny (16.1, 40.8, 45.9, 42.7, 16.0, 18.2), respectively (****p<0.0001)). (n = 23, 18, 27, 33, 24, 21). B, The ventral nerve cord from third instar larva expressing individual transgenes were dissected and metabolites were extracted for mass spectrometry. Transgenes (isoform E-3kb) were expressed under control of the pan-neuronal driver BG380-Gal4. cADPR levels were measured as a biomarker for Sarm1 NADase activity. Quantification of cADPR levels are (1.00, 8.54, 12.70, 1.75 fold, respectively) (n = 7 for each genotype). For Fig 4A and 4B, values are presented as mean +/- SEM. Statistics were obtained by performing a one-way ANOVA with Tukey’s multiple comparisons for genotypes. (**p<0.01 and ****p<0.0001).

We next investigated whether dNMNAT can block dSarm1^CE^ function for developmental signaling. Expression of dNMNAT potently blocks injury-induced axon degeneration in *Drosophila* [6,46,53,54]. Interestingly, overexpression of dNMNAT in neurons does not suppress NMJ overgrowth induced by dSarm1^CE^. Similar results were obtained upon overexpression of cyto-mNMNAT1, a cytosolic version of mammalian NMNAT1 that potently blocks axon degeneration [6] in *Drosophila* (Fig 4A). Following injury, NMNAT enzymes block SARM1 activation [49,55]. Hence, these findings suggest that dNMNAT1 may be unable to block dSarm1 activation in the developmental context. To explore this hypothesis, we tested whether expression of dNMNAT blocks activation of dSarm1^CE^ by measuring levels of the SARM1 biomarker cADPR. We expressed dSarm1^CE^ with or without co-expression of dNMNAT in neurons under the control of the pan neuronal BG380-Gal4 driver, collected ventral nerve cords from third instar larvae, and extracted metabolites for mass spectrometry (LC-MS-MS) analysis. As shown in Fig 4B, expression of dNMNAT did not suppress the dSarm1^CE^-dependent production of cADPR. The selective impact of dNMNAT expression on dSarm1-dependent injury signaling suggests that injury and developmental signaling activate dSarm1 via distinct upstream molecular mechanisms.

### Human SARM1 is functional for axon injury, but not signaling for NMJ growth

**Fig 5:**
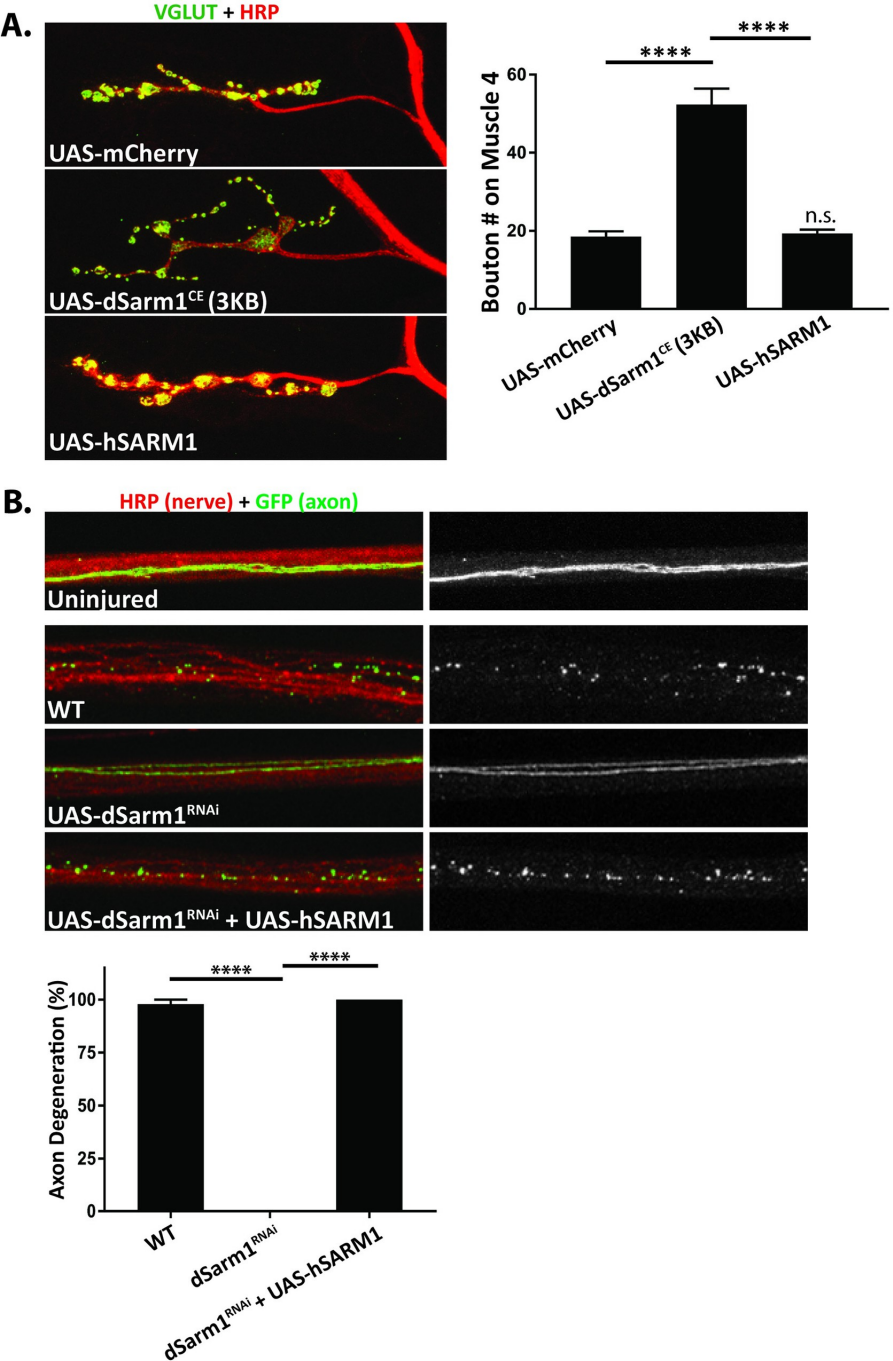
Human SARM1 functions in *Drosophila* to promote axon degeneration, but not NMJ growth. ***A*,** Overexpression of hSARM1 by dVGlut-Gal4 does not lead to NMJ overgrowth. Representative confocal images of muscle 4 synapses from third instar larva co-stained for presynaptic DVGlut (green) and neuronal HRP (Red). Quantification of the bouton number from motoneuron MN4b on muscle 4 of male progeny (18.5, 52.3, 19.3 boutons, respectively) (****p<0.0001). (n = 22, 19,22) ***B*,** Human SARM1 is activated in *Drosophila* after axonal injury. The segmental nerves of third instar larva are stained by HRP (red) and single axons are labeled by the expression of UAS-mCD8:GFP by the M12-Gal4 driver. Distal axons in wild-type larva degenerate 18 hours after axonal injury. Axons do not degenerate in axons expressing RNAi targeting endogenous dSarm1. Co-expression of human SARM1, that is resistant to the RNAi that targets endogenous dSarm1, results in degeneration of the axon. Quantification of axon degeneration of female progeny (97.9%, 0%, 100%, respectively) (****p<0.0001). For Fig 5A and 5B, values are presented as mean +/- SEM. Statistics were obtained by performing a one-way ANOVA with Tukey’s multiple comparisons. (n = 12, 12, 18).

The NADase activity of the TIR domain of human SARM1 is significantly greater than that of dSarm1 and similar to that of dSarm1^CE^ in *in vitro* enzymatic assays (Fig 1C). We asked whether the enhanced NADase activity of human SARM1 would also lead to massive NMJ overgrowth as was seen with the catalytically enhanced dSarm1^CE^. To our surprise, overexpression of human SARM1 did not lead to NMJ overgrowth (Fig 5A). It is possible that human SARM1 is not functional in *Drosophila*. Several different experiments suggested this possibility. Ubiquitous expression of human SARM1 did not rescue the lethality associated with *dsarm1* mutant flies (Table 1). This lack of rescue was not due to the enhanced NADase activity of human SARM1 leading to lethality as was seen with dSarm1^CE^ since wild-type flies expressing human SARM1 were viable (Table 1). Additionally, unlike what was seen with expression of dSarm1^CE^, pan-neuronal expression of human SARM1 did not lead to any increase in cADPR levels in the ventral nerve cord (Fig 1D). The lack of human SARM1 phenotypes was not due to a lack of expression of the transgene, since human SARM1 is easily visualized in the axon at levels similar or greater than those of the *Drosophila* transgenes (S1 Fig). To investigate whether human SARM1 is functional in *Drosophila*, we assayed its role in axon degeneration.

To test the role of human SARM1 in degeneration we performed a standard axon degeneration assay [46,56]. The segmental nerves of larvae expressing GFP in a subset of motoneurons (M12-Gal4) were crushed with fine forceps. Axon fragmentation was characterized 18 h after axonal injury (Fig 5B). In wild-type animals, the GFP-labeled axons were completely fragmented into small puncta. In contrast, in axons expressing RNAi that targets endogenous dSarm1, axons remain intact. Since the RNAi only targets *Drosophila* Sarm1, it is possible to express human SARM1 in the same neurons and assess the ability of human SARM1 to promote degeneration after injury. When human SARM1 was co-expressed in neurons with endogenous dSarm1 targeted by RNAi, human SARM1 led to robust axon degeneration. This demonstrates that human SARM1 can function in *Drosophila*, however it appears to only be activated during injury signaling (Fig 5B). Furthermore, the activation of human SARM1 requires injury since the expression of human SARM1 alone did not lead to spontaneous degeneration (S3B Fig). Taken together, these data in conjunction with prior studies show that when expressed in *Drosophila* neurons both dSARM1 and human SARM1 are activated by injury, likely due to the loss of dNmnat. In contrast, in healthy *Drosophila* neurons dSarm1 is active while human SARM1 is inactive, suggesting that the mechanism of developmental activation of dSarm1 is not conserved in the human protein.

## Discussion

**Fig 6:**
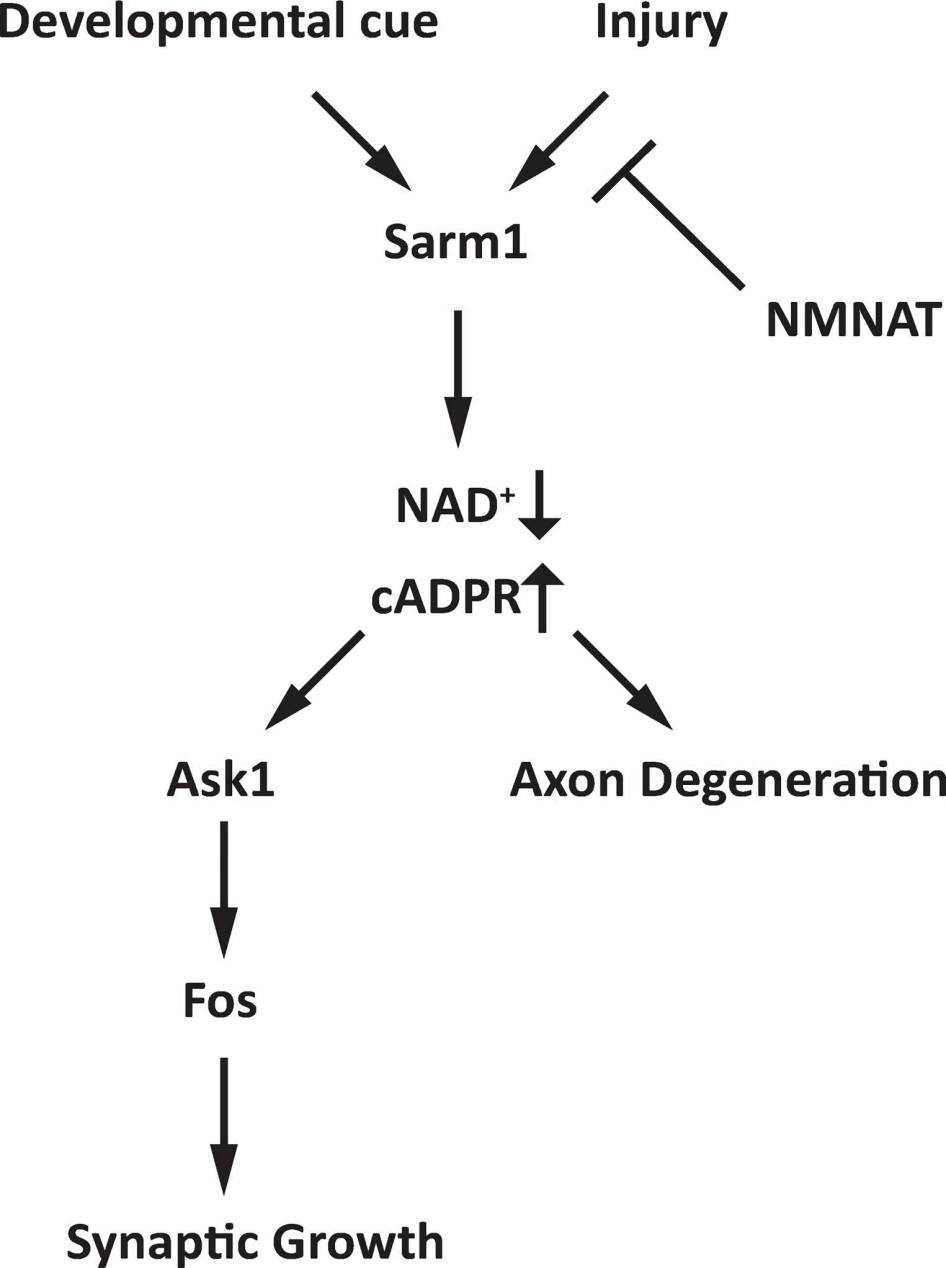
Model of dSarm1 regulation of axon degeneration and synaptic growth. Distinct upstream signals during development or after injury lead to dSarm1 activation. dSarm1 NADase activity is required for both axon degeneration and synaptic growth. The Ask1 MAPKKK pathway is necessary for dSarm1 regulation of synaptic growth, however, it is dispensable for axon degeneration.

The discovery that SARM1 is the founding member of an evolutionarily conserved family of TIR-domain NAD^+^ hydrolases defined the degenerative mechanism of SARM1 while providing new insights into the evolution and functions of TIR domains [7,25]. Here we attempted to reconcile the enzymatic activity of SARM1 with its previously described role as a regulator of signal transduction. After generating transgenic flies expressing dSarm1 with either inactive or enhanced NADase activity, we explored the previously described role of dSarm1 as a regulator of developmental signaling for the control of NMJ growth. We found that the NADase activity of dSarm1 is not only required for such signaling, but that enhanced NADase activity drives exaggerated NMJ growth. Moreover, while the role of NADase activity is shared between developmental and degenerative contexts, upstream and downstream regulatory mechanisms differ (Model in Fig 6). This study expands our understanding of SARM1 NADase activity—it is not merely an executioner of catastrophic metabolic collapse and degeneration, but also a heretofore undescribed metabolic regulator of developmental signaling. These conclusions are in excellent agreement with the independent companion paper from the Broihier lab.

### dSarm1 NADase activity functions through the Ask1 kinase pathway to regulate NMJ growth

During development, activated dSarm1 does not lead to catastrophic NAD^+^ loss, but instead functions through the Ask1 MAP kinase signal transduction pathway and the transcription factor Fos to control NMJ growth. dSarm1 and Ask1 do not regulate baseline NMJ growth, but upon activation lead to exhuberant NMJ growth. This is similar to the function of other stress proteins including the MAPKKK DLK/Wallenda at the fly NMJ, which also does not impact baseline NMJ growth but triggers dramatic overgrowth upon activation [36]. We use the term ‘signaling’ to refer to activation of Ask1 or other intracellular signal transduction pathways and not to the catastrophic NAD^+^ loss triggered during degeneration. The Ask1 pathway is selective for the regulation of NMJ growth, as Ask1 is not required for axon degeneration in *Drosophila* or mammalian DRG cultures. However, both dSarm1 and Ask1 are required for axon injury-dependent inhibition of vesicle trafficking in the fly, consistent with the hypothesis that dSarm1 may activate Ask1 for some injury-dependent phenotypes, although this activity may be NADase independent (Hsu, 2021). The SARM1-ASK1 signaling pathway was originally identified in *C*. *elegans* as a regulator of neuronal identity [29]. Although assumed to be a scaffolding molecule at the time, it is likely that tir-1, the *C*. *elegans* ortholog of SARM1, also regulates ASK1 (nsy-1) via its NADase function. In addition to the Ask1/Fos pathway described here, dSarm1 may regulate additional pathways, as the Broihier lab previously demonstrated a role for dSarm1 at the NMJ in regulating microtubules through the transcription factor FoxO [30].

We do not know how dSarm1 NADase activity regulates the Ask1 pathway, and whether it is due to decreases in NAD^+^ levels or increases in other metabolites such as ADPR or cADPR. In other systems ASK1 is regulated by the redox state of the cell. The reduced form of thioredoxin binds ASK1 and inhibits ASK1 activation, and NADPH is required to maintain thioredoxin in the reduced state [57]. We speculate that as dSarm1 cleaves NAD^+^, NADP and NADPH levels are affected and thioredoxin becomes oxidized, thereby activating Ask1. Alternatively, NAD^+^ cleavage generates cADPR and ADPR, which can each mobilize calcium [58]. Calcium, in turn, can regulate Ask1 activation via calcineurin [59]. Future studies will explore the mechanism linking dSarm1 NADase activity and Ask1 activation.

### Distinct upstream mechanisms activate dSarm1 for degenerative and developmental signaling

The activation of dSarm1 during NMJ growth regulation is distinct from its activation during injury. The mechanism of SARM1 autoinhibition and activation by injury is understood in molecular detail. SARM1 is a metabolic sensor activated by an increase in the NMN/NAD^+^ ratio [49]. In uninjured axons, the allosteric pocket of SARM1 binds NAD^+^ [60,61], while multiple intra- and intermolecular interactions in the SARM1 octamer stabilize the “off”-state of the enzyme [62]. After injury, levels of the axonal survival factor and NAD^+^ biosynthetic enzyme NMNAT2 decline [50] leading to an increase in the [NMN]/[NAD^+^] ratio. NMN then displaces NAD^+^ from the allosteric site on SARM1, destabilizing the autoinhibitory domain interactions and activating the TIR NADase activity [49]. This activation mechanism explains the potent protection provided by overexpression of NMNAT1, as NMNAT1 is a more stable protein than NMNAT2 and so can maintain a normal NMN/NAD^+^ ratio even in injured axons.

While overexpression of mammalian NMNAT1 or dNmnat potently suppress dSarm1 dependent axon degeneration, these NMNAT enzymes could not suppress the NMJ overgrowth or cADPR production due to dSarm1^CE^ overexpression. This suggests distinct mechanisms of dSarm1 activation for axon injury vs. NMJ growth. While injury activation is due to NAD^+^ metabolite changes induced by loss of endogenous dNmnat, such changes are not sufficient to block dSarm1 developmental signaling. Due to the limited amount of material it was not possible to directly test cADPR levels in the distal axons of *Drosophila* after injury. It is possible that these differences are due to higher levels of basal activation of dSarm1, although such a model would not explain why human SARM1, which is as active as dSarm1^CE^ *in vitro*, is not active in uninjured *Drosophila* neurons. Instead, we posit that protein-protein interactions or post-translational modifications likely regulate dSarm1 activation during developmental signaling. In innate immune signaling SARM1 and other TIR-domain adaptors bind to TIR domains of immune receptors forming scaffolds for additional signaling proteins. Similarily, dSarm1 may bind to and be activated by another TIR-domain containing protein such as a Toll-like receptor (TLR). Indeed, the Broihier lab found that the Toll-like receptor Toll-6 can regulate NMJ size and this function requires dSarm1 [30]. While such TIR-TIR binding is assumed to regulate scaffolding, we speculate that such heterotypic TIR-TIR interactions may activate the SARM1 NADase to regulate Ask1 activation and the downstream NMJ growth program. Alternatively, dSarm1 has long N- and C-terminal extensions compared to human SARM1, and these uncharacterized regions of the protein may be sites of regulatory protein-protein interactions or post-translational modifications.

Our findings with human SARM1 expressed in *Drosophila* also support the view that activation mechanisms for degenerative and developmental signaling are distinct. *In vitro*, the TIR domain of human SARM1 is as potent an NADase as the catalytically enhanced dSarm1^CE^. Hence, we expected that hSARM1 and dSarm1^CE^ would behave similarly in our assays. However, human SARM1 was inactive in most of our assays, failing to generate cADPR in uninjured neurons, to rescue lethality of the dSarm1 mutant, and to drive NMJ overgrowth when overexpressed. Meanwhile, human SARM1 was capable of being activated after axonal injury to drive robust axon degeneration. Our interpretation of these findings is that human SARM1 is functional in the fly and is activated by the same injury signals (NMN/NAD^+^ ratio) as dSarm1. Indeed, we have previously demonstrated structural conservation in the allosteric pocket that binds NMN and NAD^+^ between human and fly SARM1 [49]. However, we further speculate that human SARM1 is not regulated by the mechanisms activating dSarm1 for NMJ growth. This would be expected if dSarm1 is activated by protein-protein interactions or post-translational modifications, as these are less likely to be functionally conserved through evolution. Indeed, dSARM1 has long N- and C-terminal extensions that are not shared with human SARM1 and so may be the locus for this additional activation mechanism.

## Conclusion

The discovery that SARM1 is an NADase defined the fundamental molecular mechanism of pathological axon degeneration. Here, we show that dSarm1 NADase activity also regulates intracellular signal transduction and is required for survival during *Drosophila* development. By analogy, our findings suggest that SARM1-dependent regulation of NAD^+^ metabolism may be the mechanism by which SARM1 influences innate immune signaling [63]. SARM1 enzyme inhibitors are being developed as potential therapeutics for the maintenance of axons in the injured and diseased nervous systems [64–66]. If SARM1 NADase activity also regulates mammalian signal transduction, then it will be interesting to assess the influence of such SARM1 inhibitors on the relevant signaling pathways.

## Materials and methods

### Ethics statement

All animal experiments were performed in accordance with the policies and guidelines of the Institutional Animal Care and Use Committee (IACUC) of Washington University in St. Louis (specific protocols #20–0020 and #20–0484).

*Fly stocks*. *Drosophila melanogaster* were raised on standard fly food at 25°C. The following strains were used in this study: m12-Gal4 [67], *dVGlut*-Gal4 [68], BG380-Gal4 [69], UAS-cyto-mNMNAT1-mCherry [6], UAS-Fos^D/N^ [70], sarm^4705^ and sarm^4621^ [3], puc-LacZ^E69^ [44]. RNAi lines were obtained from the Bloomington Stock Center (BL) or Vienna (V) stock center: UAS-Sarm RNAi^KK105369^ (V), UAS-Wnd-RNAi^103410^ (V), UAS-NMNAT RNAi^107262^ (V), UAS-LexA RNAi^67947^ (BL), UAS-Ask1^RNA#1^ (Vienna KK110228), UAS-Ask1^RNA#2^ (BL 35331). Additional lines obtained from stock centers include: UAS-CD8-GFP (BL 5139), UAS-mCherry (BL 32218), UAS-dNmnat-HA (BL 39702), *daughterless*-Gal4 (BL 55850), UAS-Dcr2 (BL 24650). Gal4 lines that were lethal with dSarm1^CE^ included: *Da*-gal4 (BL 55850), *69B*-gal4 (BL1774), *24B*-gal4 (BL1767), *elav*-gal4 [71], and *repo*-gal4 [72]. The cDNA for *Drosophila* isoform D of dSarm1 (5kb) was obtained from the pUAST-dSarm1 construct (Freeman lab) [3]. The cDNA for *Drosophila* isoform E of dSarm1 (3kb) was obtained from the DGRC (GH20978). Human SARM1 cDNA was obtained from OpenBiosystems [4]. The cDNAs were cloned into pUAST-attB with a C-terminal HA-tag. Transgenics were created by Best Gene with the landing site strain ZH-86Fb (BDSC#24749). The catalytically enhance (CE) mutation is equivalent to the E604 amino acid in human SARM1 (D_855_E in 3 kb isoform or D_1252_E in 5 kb isoform). The enzymatic dead mutation is equivalent to E_642_A in human SARM1 (E_893_A in 3 kb isoform or E_1290_A in 5 kb isoform).

### Immunocytochemistry

Larval filet preps were fixed in Bouin’s solution (a 1:5:15 ratio of acetic acid/formalin/picric acid) for 10 minutes at room temperature (VGLUT staining) or 4% paraformaldehyde in PBS for 20 minutes at room temperature (GFP staining) [56]. Blocking and staining were performed in PBS + 0.1% Triton X-100 +5% goat serum. The rabbit α-DVGLUT antibody was described previously (Daniels et al. [68]), and used at 1:5,000 dilution. Additionally, rat α-Elav (1:50, Developmental Studies Hybridoma Bank AB_52818; mouse α-beta galactosidase (1:100, Developmental Studies Hybridoma Bank AB_2314509). Other antibodies were: Cy3 and Cy5 conjugated goat α-HRP (1:1000, Jackson ImmunoResearch), Alexa 488 conjugated α-rabbit (1:1000, Molecular Probes) and Cy3 α-mouse (1:1000, Jackson ImmunoResearch), Alexa 488 conjugated Rabbit αGFP (1:1000 Life Technologies), AlexaFluor 488 chicken α-rat (1:1000, ThermosScientific-Invitrogen Cat# A-21470), Cy3 goat α-mouse (1:1000, Jackson ImmunoResearch, Cat#115-165-146), anti-HA (C29F4 Rabbit mAb, 1:1500 Cell Signaling). All samples were mounted and imaged in 70% glycerol containing Vectashield.

### Nerve crush assay

The segmental nerves of third instar larva were visualized on the ventral side of the larvae under a dissection microscope. Larvae were immobilized in a cold dissecting dish and the nerves were pinched through the cuticle with #5 forceps. Injured larvae were transferred to a grape plate with yeast paste for 18 hours at 25°C to allow time for axon degeneration. This assay was adapted from [38,56]. Axons were labeled with CD8-GFP expressed in single axons by the M12-Gal4 driver. Dissected larvae were fixed in PBS + 4% paraformaldehyde for 20 minutes at room temperature and washed in PBS + 0.1% Triton. Larvae were stained with α-GFP (1:1000) and Cy3-αHRP (1:1000) to visualize nerves and individual axons. Degeneration scoring index was adapted from [38].

### Imaging and analysis

Images of *Drosophila* third instar larva were acquired on a Leica DMI4000B Confocal microscope using 40x or 60x oil objectives. Images shown are maximal Z-projections of confocal stacks. Samples for each experiment were processed simultaneously and used identical confocal gain settings and laser power. Bouton numbers were scored manually from muscle 4 synapses while blinded to genotype. Boutons were defined as distinct puncta of dVGLUT staining. Statistics were obtained by performing one-way ANOVA with Tukey’s multiple comparisons for genotypes. The mean intensity of LacZ expression was quantified in image J by measuring the intensity of LacZ staining in the neuronal nuclei that lie along the dorsal midline of the nerve cord. Elav staining was used to identify the neurons and create the subsequent mask used for quantification of LacZ. Intensity was normalized to wild-type controls. Statistics obtained by performing a one-way ANOVA with Tukey’s multiple comparisons for genotypes.

### TIR proteins expression and purification

The protocol for protein expression and purification was described previously [7]. The TIR domains were cloned into FCIV containing an N-terminal Strep-tag and a C-term Venus tag. TIR proteins were expressed in NRK1-HEK293T cells in the presence of nicotinamide riboside (NR) (1mM). Cells were lysed by sonication and followed by one step purification with 100μl MagStrep (Strep-Tactin) type 3 XT beads suspension (IBA Lifesciences).

### NADase assay and HPLC measurements

The protocol for NADase assay and HPLC metabolites measurements were described previously [7]. One hundred nanograms of TIR proteins (2–5μl beads) were used for NADase assays with 5μM NAD^+^ in 20μL reaction. Reactions were carried out at 37°C for the indicated time and stopped by addition of 1M of perchloric acid (HClO_4_). NAD^+^ metabolites were extracted using HClO_4_/K_2_CO_3_ method. Extracted metabolites were analyzed by HPLC (Nexera 2) with Kinetex (100 x 3mm, 2.6μm; Phenomenex) column. The experiments were repeated three times.

### Metabolite extraction from Drosophila brain for mass spectrometry

The ventral nerve cord from third instar larvae were dissected (5 brains per genotype were pooled n = 1) in a microcentrifuge tube and stored at -80°C. On the day of extraction, tissues were placed on ice, mixed with 100 μl of cold 50% methanol (Thermo Fisher Scientific) in water, and homogenized by the sonication (Branson, Sonifier 450, output 1.5, 50% duration, 10 seconds). Samples were centrifuged (20,000 g, 10 min, 4°C) and the cleared supernatant (100 μl) was transferred to a new tube containing 30 μl chloroform (Sigma). Precipitated tissues were kept for protein quantification as described below. The tubes containing metabolites and chloroform were shaken vigorously and then centrifuged (20,000 g, 10 min, 4°C). The clear aqueous phase (80 μl) was transferred to a microfuge tube and lyophilized under the vacuum. Lyophilized samples were stored at -20°C until measurement. For protein quantification, precipitated tissues were homogenized in 0.1% SDS in water (100 μl) with sonication (output 1.5, 50% duration, 10 seconds), centrifuged (20,000 g, 5 min, 4°C), and protein concentration of the supernatant was measured using BCA protein assay (Thermo Fisher Scientific).

### Metabolite measurements from mass spectrometry

Lyophilized samples were reconstituted with 5 mM ammonium formate (Sigma, 15 μl), centrifuged (20,000 *g*, 10 min, 4°C), and 10 μl of the cleared supernatant was analyzed using mass spectroscopy [55,73]. Metabolites were separated using HPLC (Agilent: 1290 LC) equipped with a C18 reverse phase column (Atlantis T3, 2.1 x 150 mm, 3 μm; Waters) at a flow rate of 0.15 ml/min with 5 mM ammonium formate for mobile buffer A. Samples were eluted with gradients of mobile buffer B (100% methanol) as 0–10 min, 0–70% B; 10–15 min, 70% B; 16–20 min, 0% B and monitored with a triple quad mass spectrometer (Agilent 6470) under a positive ESI multiple reaction monitoring (MRM) mode using parameters specific for each compound (NAD^+^ (Sigma), 664>428, fragmentation (F) = 160 V, collision (C) = 22 V, and cell acceleration (CA) = 7 V: cADPR (Sigma), 542>428, F = 100 V, C = 20 V, and CA = 3 V). Serial dilutions of standards for each metabolite in 5 mM ammonium formate were used for calibration. Metabolites were quantified by quantitative analysis tool (Agilent, MassHunter) with standard curves and normalized by the protein.

### Axon degeneration of mouse primary embryonic DRG neurons

Embryonic DRG spot cultures were prepared as previously described [55]. In brief, DRGs were isolated from embryonic day 12.5–13.5 wild-type CD1 pregnant mice (Charles River Laboratories (Wilmington, MA)) and cultured on plates coated with poly-D-lysine and laminin. Neurobasal culture medium (Thermo-Fisher(Waltham,MA); Cat.#21103049) was supplemented with 2% B27, 50ng/mL nerve growth factor (Envigo Bioproducts (Indianapolis, IN) Cat.#B5017), and 10uM 5-fluoro-2’-deoxyuridine (Sigma Aldrich (St. Louis, MO); Cat.#F0503) and 10uM uridine (Sigma-Aldrich (St. Louis, MO); Cat.#U3003). On DIV4, half the media was removed and replaced with new media. All experiments were performed at DIV6-7. Selonsertib (GS-4997) was obtained from Selleck Chemicals (Houston, TX (Cat.#S8292)). Selonsertib was dissolved in DMSO at 10mM and stored at -20°C. DRG spot cultures in 24-well plate were preincubated with 1.5uM Selonsertib for 1hr and 24hr prior to axotomy. Briefly, axons from DRG spot cultures in 24-well plates were transected using a microsurgical blade under a microscope at DIV7. Bright-field images of distal axons (20 fields/well and 2 wells/condition) were taken at 0 and 16 h after axotomy with a 20x objective [74]. Axon images were collected with an Operetta High Content Image System (PerkinElmer) and their degree of fragmentation was quantified using an in-house ImageJ macro as previously described [55,74].

## Supporting information

S1 Fig Sarm1 transgene levels. A. Sarm1-HA tagged transgenes were expressed under control of the dVGlut-Gal4 driver. The segmental nerves of third instar larvae are shown visualizing HA-tagged dSarm1 (anti-HA antibody: C29F4 1:1500 dilution). Images of transgenes from the same class (isoform E- 3KB or isoform D- 5KB) were taken at similar levels. B. Quantification of transgene levels. Quantification of images for all genotypes were taken at the same gain and intensity settings. Values are presented as mean +/- standard deviation (n = 4, 4, 4, 5, 5, 5, 4, 5, 5 for each genotype). Statistics obtained by performing a one-way ANOVA with Tukey’s multiple comparisons for genotypes (****p<0.0001). The mean intensity of fluorescence was quantified in image J.
(EPS)

S2 Fig Ask1 inhibitor does not slow axon degeneration in axotomy of mammalian DRG axons. DRG neuron cultures were pretreated with the Ask1 inhibitor (Selonsertib) prior to axotomy. 16 hours after axotomy the distal axons from inhibitor treated neurons degenerated similar to untreated axons as measured by the degeneration index (n = 3). Values are presented as mean ± SEM. P value: ****≤ 0.0001 by ANOVA with Tukey’s multiple comparisons.
(EPS)

S3 Fig The dSarm1^CE^ allele is regulated by dNMNAT. **A.** The segmental nerves of third instar larva are stained by HRP (red) and single axons are labeled by the expression of UAS-mCD8:GFP by the M12-Gal4 driver. Knockdown of dNMNAT leads to spontaneous axon degeneration in *dsarm1* heterozygous animals. This degeneration is blocked in homozygous *dsarm1* mutant animals. Reexpression of dSarm1^CE^ (isoform E -3kb) in *dsarm1* mutant animals result in robust axon degeneration upon dNMNAT knockdown. Thus demonstrating that the gain of function allele maintains regulation by NMNAT. **B.**, Expression of Sarm1 transgenes do not result in spontaneous degeneration. The segmental nerves of third instar larva are stained by HRP (red) and single axons are labeled by the expression of UAS-mCD8:GFP by the M12-Gal4 driver. Labeled axons are also expressing Sarm1 transgenes. In the absence of injury, axons do not spontaneously degenerate due to the expression of hSARM1 or dSarm1^CE^.
(EPS)

S1 Data Excel graph with the data that underlie graphs throughout manuscript. (XLSX)

## References

[1] Figley MD, DiAntonio A. The SARM1 axon degeneration pathway: control of the NAD+ metabolome regulates axon survival in health and disease. Curr Opin Neurobiol. 2020;63: 59–66. doi: 10.1016/j.conb.2020.02.012 32311648 PMC7483800

[2] Coleman MP, Höke A. Programmed axon degeneration: from mouse to mechanism to medicine. Nat Rev Neurosci. 2020; 1–14. doi: 10.1038/s41583-020-0269-3 32152523 PMC8926152

[3] Osterloh JM, Yang J, Rooney TM, Fox AN, Adalbert R, Powell EH, et al. dSarm/Sarm1 Is Required for Activation of an Injury-Induced Axon Death Pathway. Science. 2012;337: 481–484. doi: 10.1126/science.1223899 22678360 PMC5225956

[4] Gerdts J, Summers DW, Sasaki Y, DiAntonio A, Milbrandt J. Sarm1-Mediated Axon Degeneration Requires Both SAM and TIR Interactions. J Neurosci. 2013;33: 13569–13580. doi: 10.1523/JNEUROSCI.1197-13.2013 23946415 PMC3742939

[5] Ko KW, Devault L, Sasaki Y, Milbrandt J, DiAntonio A. Live imaging reveals the cellular events downstream of SARM1 activation. Elife. 2021;10: e71148. doi: 10.7554/eLife.71148 34779400 PMC8612704

[6] Gerdts J, Brace EJ, Sasaki Y, DiAntonio A, Milbrandt J. SARM1 activation triggers axon degeneration locally via NAD^+^ destruction. Science. 2015;348: 453–457. doi: 10.1126/science.1258366 25908823 PMC4513950

[7] Essuman K, Summers DW, Sasaki Y, Mao X, DiAntonio A, Milbrandt J. The SARM1 Toll/Interleukin-1 Receptor Domain Possesses Intrinsic NAD+ Cleavage Activity that Promotes Pathological Axonal Degeneration. Neuron. 2017;93: 1334–1343.e5. doi: 10.1016/j.neuron.2017.02.022 28334607 PMC6284238

[8] Chen Y-H, Sasaki Y, DiAntonio A, Milbrandt J. SARM1 is required in human derived sensory neurons for injury-induced and neurotoxic axon degeneration. Exp Neurol. 2021;339: 113636. doi: 10.1016/j.expneurol.2021.113636 33548217 PMC8171232

[9] Krauss R, Bosanac T, Devraj R, Engber T, Hughes RO. Axons Matter: The Promise of Treating Neurodegenerative Disorders by Targeting SARM1-Mediated Axonal Degeneration. Trends Pharmacol Sci. 2020;41: 281–293. doi: 10.1016/j.tips.2020.01.006 32107050

[10] Geisler S, Doan RA, Strickland A, Huang X, Milbrandt J, DiAntonio A. Prevention of vincristine-induced peripheral neuropathy by genetic deletion of SARM1 in mice. Brain. 2016;139: 3092–3108. doi: 10.1093/brain/aww251 27797810 PMC5840884

[11] Geisler S, Doan RA, Cheng GC, Cetinkaya-Fisgin A, Huang SX, Höke A, et al. Vincristine and bortezomib use distinct upstream mechanisms to activate a common SARM1-dependent axon degeneration program. Jci Insight. 2019;4: e129920. doi: 10.1172/jci.insight.129920 31484833 PMC6777905

[12] Turkiew E, Falconer D, Reed N, Höke A. Deletion of Sarm1 gene is neuroprotective in two models of peripheral neuropathy. J Peripher Nerv Syst. 2017;22: 162–171. doi: 10.1111/jns.12219 28485482 PMC5585053

[13] Cheng Y, Liu J, Luan Y, Liu Z, Lai H, Zhong W, et al. Sarm1 Gene Deficiency Attenuates Diabetic Peripheral Neuropathy in Mice. Diabetes. 2019;68: 2120–2130. doi: 10.2337/db18-1233 31439642 PMC6804630

[14] Marion CM, McDaniel DP, Armstrong RC. Sarm1 deletion reduces axon damage, demyelination, and white matter atrophy after experimental traumatic brain injury. Exp Neurol. 2019;321: 113040. doi: 10.1016/j.expneurol.2019.113040 31445042

[15] Henninger N, Bouley J, Sikoglu EM, An J, Moore CM, King JA, et al. Attenuated traumatic axonal injury and improved functional outcome after traumatic brain injury in mice lacking Sarm1. Brain. 2016;139: 1094–1105. doi: 10.1093/brain/aww001 26912636 PMC5006226

[16] Maynard ME, Redell JB, Zhao J, Hood KN, Vita SM, Kobori N, et al. Sarm1 loss reduces axonal damage and improves cognitive outcome after repetitive mild closed head injury. Exp Neurol. 2020;327: 113207. doi: 10.1016/j.expneurol.2020.113207 31962129 PMC7959192

[17] Ko KW, Milbrandt J, DiAntonio A. SARM1 acts downstream of neuroinflammatory and necroptotic signaling to induce axon degeneration. J Cell Biol. 2020;219: e201912047. doi: 10.1083/jcb.201912047 32609299 PMC7401797

[18] Sasaki Y, Kakita H, Kubota S, Sene A, Lee TJ, Ban N, et al. SARM1 depletion rescues NMNAT1-dependent photoreceptor cell death and retinal degeneration. Elife. 2020;9: e62027. doi: 10.7554/eLife.62027 33107823 PMC7591247

[19] Ozaki E, Gibbons L, Neto NG, Kenna P, Carty M, Humphries M, et al. SARM1 deficiency promotes rod and cone photoreceptor cell survival in a model of retinal degeneration. Life Sci Alliance. 2020;3: e201900618. doi: 10.26508/lsa.201900618 32312889 PMC7184027

[20] Bloom AJ, Mao X, Strickland A, Sasaki Y, Milbrandt J, DiAntonio A. Constitutively active SARM1 variants that induce neuropathy are enriched in ALS patients. Mol Neurodegener. 2022;17: 1. doi: 10.1186/s13024-021-00511-x 34991663 PMC8739729

[21] Gilley J, Jackson O, Pipis M, Estiar MA, Al-Chalabi A, Danzi MC, et al. Enrichment of SARM1 alleles encoding variants with constitutively hyperactive NADase in patients with ALS and other motor nerve disorders. Elife. 2021;10: e70905. doi: 10.7554/eLife.70905 34796871 PMC8735862

[22] Luo L, Lucas RM, Liu L, Stow JL. Signalling, sorting and scaffolding adaptors for Toll-like receptors. J Cell Sci. 2020;133: jcs239194. doi: 10.1242/jcs.239194 31889021

[23] Ve T, Williams SJ, Kobe B. Structure and function of Toll/interleukin-1 receptor/resistance protein (TIR) domains. Apoptosis. 2015;20: 250–261. doi: 10.1007/s10495-014-1064-2 25451009

[24] Horsefield S, Burdett H, Zhang X, Manik MK, Shi Y, Chen J, et al. NAD+ cleavage activity by animal and plant TIR domains in cell death pathways. Science. 2019;365: 793–799. doi: 10.1126/science.aax1911 31439792

[25] Essuman K, Summers DW, Sasaki Y, Mao X, Yim AKY, DiAntonio A, et al. TIR Domain Proteins Are an Ancient Family of NAD+-Consuming Enzymes. Curr Biol. 2018;28: 421–430.e4. doi: 10.1016/j.cub.2017.12.024 29395922 PMC5802418

[26] Wan L, Essuman K, Anderson RG, Sasaki Y, Monteiro F, Chung E-H, et al. TIR domains of plant immune receptors are NAD+-cleaving enzymes that promote cell death. Science. 2019;365: 799–803. doi: 10.1126/science.aax1771 31439793 PMC7045805

[27] Carty M, Goodbody R, Schröder M, Stack J, Moynagh PN, Bowie AG. The human adaptor SARM negatively regulates adaptor protein TRIF–dependent Toll-like receptor signaling. Nat Immunol. 2006;7: 1074–1081. doi: 10.1038/ni1382 16964262

[28] Couillault C, Pujol N, Reboul J, Sabatier L, Guichou J-F, Kohara Y, et al. TLR-independent control of innate immunity in Caenorhabditis elegans by the TIR domain adaptor protein TIR-1, an ortholog of human SARM. Nat Immunol. 2004;5: 488–494. doi: 10.1038/ni1060 15048112

[29] Chuang C-F, Bargmann CI. A Toll-interleukin 1 repeat protein at the synapse specifies asymmetric odorant receptor expression via ASK1 MAPKKK signaling. Gene Dev. 2005;19: 270–281. doi: 10.1101/gad.1276505 15625192 PMC545892

[30] McLaughlin CN, Nechipurenko IV, Liu N, Broihier HT. A Toll receptor–FoxO pathway represses Pavarotti/MKLP1 to promote microtubule dynamics in motoneuronsA Toll receptor pathway promotes neuronal MT dynamics. J Cell Biology. 2016;214: 459–474. doi: 10.1083/jcb.201601014 27502486 PMC4987293

[31] McLaughlin CN, Perry-Richardson JJ, Coutinho-Budd JC, Broihier HT. Dying Neurons Utilize Innate Immune Signaling to Prime Glia for Phagocytosis during Development. Dev Cell. 2019;48: 506–522.e6. doi: 10.1016/j.devcel.2018.12.019 30745142 PMC6394877

[32] Hsu J-M, Kang Y, Corty MM, Mathieson D, Peters OM, Freeman MR. Injury-Induced Inhibition of Bystander Neurons Requires dSarm and Signaling from Glia. Neuron. 2020. doi: 10.1016/j.neuron.2020.11.012 33296670 PMC7864878

[33] Herrmann KA, Liu Y, Llobet-Rosell A, McLaughlin CN, Neukomm LJ, Coutinho-Budd JC, et al. Divergent signaling requirements of dSARM in injury-induced degeneration and developmental glial phagocytosis. PLoS Genet. 2022;18(6): e1010257. doi: 10.1371/journal.pgen.1010257 PMC9223396 35737721

[34] Sasaki Y, Engber TM, Hughes RO, Figley MD, Wu T, Bosanac T, et al. cADPR is a gene dosage-sensitive biomarker of SARM1 activity in healthy, compromised, and degenerating axons. Exp Neurol. 2020; 113252. doi: 10.1016/j.expneurol.2020.113252 32087251 PMC7302925

[35] Neukomm LJ, Burdett TC, Seeds AM, Hampel S, Coutinho-Budd JC, Farley JE, et al. Axon Death Pathways Converge on Axundead to Promote Functional and Structural Axon Disassembly. Neuron. 2017;95: 78–91.e5. doi: 10.1016/j.neuron.2017.06.031 28683272

[36] Collins CA, Wairkar YP, Johnson SL, DiAntonio A. Highwire Restrains Synaptic Growth by Attenuating a MAP Kinase Signal. Neuron. 2006;51: 57–69. doi: 10.1016/j.neuron.2006.05.026 16815332

[37] Li J, Zhang YV, Adib EA, Stanchev DT, Xiong X, Klinedinst S, et al. Restraint of presynaptic protein levels by Wnd/DLK signaling mediates synaptic defects associated with the kinesin-3 motor Unc-104. Elife. 2017;6: e24271. doi: 10.7554/eLife.24271 28925357 PMC5605197

[38] Xiong X, Wang X, Ewanek R, Bhat P, DiAntonio A, Collins CA. Protein turnover of the Wallenda/DLK kinase regulates a retrograde response to axonal injury. J Cell Biol. 2010;191: 211–223. doi: 10.1083/jcb.201006039 20921142 PMC2953441

[39] Yang J, Wu Z, Renier N, Simon DJ, Uryu K, Park DS, et al. Pathological Axonal Death through a MAPK Cascade that Triggers a Local Energy Deficit. Cell. 2015;160: 161–176. doi: 10.1016/j.cell.2014.11.053 25594179 PMC4306654

[40] Etter PD, Narayanan R, Navratilova Z, Patel C, Bohmann D, Jasper H, et al. Synaptic and genomic responses to JNK and AP-1 signaling in Drosophila neurons. Bmc Neurosci. 2005;6: 39. doi: 10.1186/1471-2202-6-39 15932641 PMC1175850

[41] Nakata K, Abrams B, Grill B, Goncharov A, Huang X, Chisholm AD, et al. Regulation of a DLK-1 and p38 MAP Kinase Pathway by the Ubiquitin Ligase RPM-1 Is Required for Presynaptic Development. Cell. 2005;120: 407–420. doi: 10.1016/j.cell.2004.12.017 15707898

[42] Valakh V, Walker LJ, Skeath JB, DiAntonio A. Loss of the Spectraplakin Short Stop Activates the DLK Injury Response Pathway in Drosophila. J Neurosci. 2013;33: 17863–17873. doi: 10.1523/JNEUROSCI.2196-13.2013 24198375 PMC3818558

[43] Collins CA, DiAntonio A. Synaptic development: insights from Drosophila. Curr Opin Neurobiol. 2007;17: 35–42. doi: 10.1016/j.conb.2007.01.001 17229568

[44] Martín-Blanco E, Gampel A, Ring J, Virdee K, Kirov N, Tolkovsky AM, et al. puckered encodes a phosphatase that mediates a feedback loop regulating JNK activity during dorsal closure in Drosophila. Gene Dev. 1998;12: 557–570. doi: 10.1101/gad.12.4.557 9472024 PMC316530

[45] Brace EJ, Wu C, Valakh V, DiAntonio A. SkpA Restrains Synaptic Terminal Growth during Development and Promotes Axonal Degeneration following Injury. J Neurosci. 2014;34: 8398–8410. doi: 10.1523/JNEUROSCI.4715-13.2014 24948796 PMC4061385

[46] Xiong X, Collins CA. A Conditioning Lesion Protects Axons from Degeneration via the Wallenda/DLK MAP Kinase Signaling Cascade. J Neurosci. 2012;32: 610–615. doi: 10.1523/JNEUROSCI.3586-11.2012 22238096 PMC3280217

[47] Lu M, Yan X-F, Si Y, Chen X-Z. CTGF Triggers Rat Astrocyte Activation and Astrocyte-Mediated Inflammatory Response in Culture Conditions. Inflammation. 2019;42: 1693–1704. doi: 10.1007/s10753-019-01029-7 31183597 PMC6717176

[48] Mukherjee S, Zhelnin L, Sanfiz A, Pan J, Li Z, Yarde M, et al. Development and validation of an in vitro 3D model of NASH with severe fibrotic phenotype. Am J Transl Res. 2019;11: 1531–1540. 30972180 PMC6456529

[49] Figley MD, Gu W, Nanson JD, Shi Y, Sasaki Y, Cunnea K, et al. SARM1 is a metabolic sensor activated by an increased NMN/NAD+ ratio to trigger axon degeneration. Neuron. 2021;109: 1118–1136.e11. doi: 10.1016/j.neuron.2021.02.009 33657413 PMC8174188

[50] Gilley J, Coleman MP. Endogenous Nmnat2 Is an Essential Survival Factor for Maintenance of Healthy Axons. Plos Biol. 2010;8: e1000300. doi: 10.1371/journal.pbio.1000300 20126265 PMC2811159

[51] Xiong X, Hao Y, Sun K, Li J, Li X, Mishra B, et al. The Highwire Ubiquitin Ligase Promotes Axonal Degeneration by Tuning Levels of Nmnat Protein. Plos Biol. 2012;10: e1001440. doi: 10.1371/journal.pbio.1001440 23226106 PMC3514318

[52] Araki T, Sasaki Y, Milbrandt J. Increased Nuclear NAD Biosynthesis and SIRT1 Activation Prevent Axonal Degeneration. Science. 2004;305: 1010–1013. doi: 10.1126/science.1098014 15310905

[53] MacDonald JM, Beach MG, Porpiglia E, Sheehan AE, Watts RJ, Freeman MR. The Drosophila Cell Corpse Engulfment Receptor Draper Mediates Glial Clearance of Severed Axons. Neuron. 2006;50: 869–881. doi: 10.1016/j.neuron.2006.04.028 16772169

[54] Hoopfer ED, McLaughlin T, Watts RJ, Schuldiner O, O’Leary DDM, Luo L. Wlds Protection Distinguishes Axon Degeneration following Injury from Naturally Occurring Developmental Pruning. Neuron. 2006;50: 883–895. doi: 10.1016/j.neuron.2006.05.013 16772170

[55] Sasaki Y, Nakagawa T, Mao X, DiAntonio A, Milbrandt J. NMNAT1 inhibits axon degeneration via blockade of SARM1-mediated NAD+ depletion. Elife. 2016;5: e19749. doi: 10.7554/eLife.19749 27735788 PMC5063586

[56] Brace EJ, DiAntonio A. Axon Degeneration, Methods and Protocols. Methods Mol Biology. 2020;2143: 311–320. doi: 10.1007/978-1-0716-0585-1_23 32524490

[57] Katagiri K, Matsuzawa A, Ichijo H. Chapter 16 Regulation of Apoptosis Signal-Regulating Kinase 1 in Redox Signaling. Methods Enzymol. 2010;474: 277–288. doi: 10.1016/S0076-6879(10)74016-7 20609916

[58] Guse AH. Calcium mobilizing second messengers derived from NAD. Biochimica Et Biophysica Acta Bba—Proteins Proteom. 2015;1854: 1132–1137. doi: 10.1016/j.bbapap.2014.12.015 25534250

[59] Guo L, Urban JF, Zhu J, Paul WE. Elevating Calcium in Th2 Cells Activates Multiple Pathways to Induce IL-4 Transcription and mRNA Stabilization. J Immunol. 2008;181: 3984–3993. doi: 10.4049/jimmunol.181.6.3984 18768853 PMC2744309

[60] Jiang Y, Liu T, Lee C-H, Chang Q, Yang J, Zhang Z. The NAD+-mediated self-inhibition mechanism of pro-neurodegenerative Sarm1. Nature. 2020; 1–9. doi: 10.1038/s41586-020-2862-z 33053563

[61] Sporny M, Guez-Haddad J, Khazma T, Yaron A, Dessau M, Shkolnisky Y, et al. The structural basis for SARM1 inhibition and activation under energetic stress. Elife. 2020;9: e62021. doi: 10.7554/eLife.62021 33185189 PMC7688312

[62] Shen C, Vohra M, Zhang P, Mao X, Figley MD, Zhu J, et al. Multiple domain interfaces mediate SARM1 autoinhibition. Proc National Acad Sci. 2021;118: e2023151118. doi: 10.1073/pnas.2023151118 33468661 PMC7848697

[63] Carty M, Bowie AG. SARM: From immune regulator to cell executioner. Biochem Pharmacol. 2019;161: 52–62. doi: 10.1016/j.bcp.2019.01.005 30633870

[64] Hughes RO, Bosanac T, Mao X, Engber TM, DiAntonio A, Milbrandt J, et al. Small Molecule SARM1 Inhibitors Recapitulate the SARM1−/− Phenotype and Allow Recovery of a Metastable Pool of Axons Fated to Degenerate. Cell Reports. 2021;34: 108588. doi: 10.1016/j.celrep.2020.108588 33406435 PMC8179325

[65] Bosanac T, Hughes RO, Engber T, Devraj R, Brearley A, Danker K, et al. Pharmacological SARM1 inhibition protects axon structure and function in paclitaxel-induced peripheral neuropathy. Brain. 2021; awab184–. doi: 10.1093/brain/awab184 33964142 PMC8634121

[66] Geisler S, Huang SX, Strickland A, Doan RA, Summers DW, Mao X, et al. Gene therapy targeting SARM1 blocks pathological axon degeneration in mice. J Exp Medicine. 2019;216: 294–303. doi: 10.1084/jem.20181040 30642945 PMC6363435

[67] Ritzenthaler S, Suzuki E, Chiba A. Postsynaptic filopodia in muscle cells interact with innervating motoneuron axons. Nat Neurosci. 2000;3: 1012–1017. doi: 10.1038/79833 11017174

[68] Daniels RW, Gelfand MV, Collins CA, DiAntonio A. Visualizing glutamatergic cell bodies and synapses in Drosophila larval and adult CNS. J Comp Neurol. 2008;508: 131–152. doi: 10.1002/cne.21670 18302156

[69] Budnik V, Koh Y-H, Guan B, Hartmann B, Hough C, Woods D, et al. Regulation of Synapse Structure and Function by the Drosophila Tumor Suppressor Gene dlg. Neuron. 1996;17: 627–640. doi: 10.1016/s0896-6273(00)80196-8 8893021 PMC4661176

[70] Ciapponi L, Jackson DB, Mlodzik M, Bohmann D. Drosophila Fos mediates ERK and JNK signals via distinct phosphorylation sites. Gene Dev. 2001;15: 1540–1553. doi: 10.1101/gad.886301 11410534 PMC312716

[71] Yao K, White K. Neural Specificity of elav Expression: Defining a Drosophila Promoter for Directing Expression to the Nervous System. J Neurochem. 1994;63: 41–51. doi: 10.1046/j.1471-4159.1994.63010041.x 8207445

[72] Sepp KJ, Schulte J, Auld VJ. Peripheral Glia Direct Axon Guidance across the CNS/PNS Transition Zone. Dev Biol. 2001;238: 47–63. doi: 10.1006/dbio.2001.0411 11783993

[73] Hikosaka K, Ikutani M, Shito M, Kazuma K, Gulshan M, Nagai Y, et al. Deficiency of Nicotinamide Mononucleotide Adenylyltransferase 3 (Nmnat3) Causes Hemolytic Anemia by Altering the Glycolytic Flow in Mature Erythrocytes*. J Biol Chem. 2014;289: 14796–14811. doi: 10.1074/jbc.M114.554378 24739386 PMC4031534

[74] Gerdts J, Sasaki Y, Vohra B, Marasa J, Milbrandt J. Image-based Screening Identifies Novel Roles for IκB Kinase and Glycogen Synthase Kinase 3 in Axonal Degeneration. J Biol Chem. 2011;286: 28011–28018. doi: 10.1074/jbc.M111.250472 21685387 PMC3151046

